# RAPIDSNPs: A new computational pipeline for rapidly identifying key genetic variants reveals previously unidentified SNPs that are significantly associated with individual platelet responses

**DOI:** 10.1371/journal.pone.0175957

**Published:** 2017-04-25

**Authors:** Bajuna Rashid Salehe, Chris Ian Jones, Giuseppe Di Fatta, Liam James McGuffin

**Affiliations:** 1 School of Biological Sciences, University of Reading, Reading, United Kingdom; 2 Department of Computer Science, University of Reading, Reading, United Kingdom; Centro Cardiologico Monzino, ITALY

## Abstract

Advances in omics technologies have led to the discovery of genetic markers, or single nucleotide polymorphisms (SNPs), that are associated with particular diseases or complex traits. Although there have been significant improvements in the approaches used to analyse associations of SNPs with disease, further optimised and rapid techniques are needed to keep up with the rate of SNP discovery, which has exacerbated the ‘missing heritability’ problem. Here, we have devised a novel, integrated, heuristic-based, hybrid analytical computational pipeline, for rapidly detecting novel or key genetic variants that are associated with diseases or complex traits. Our pipeline is particularly useful in genetic association studies where the genotyped SNP data are highly dimensional, and the complex trait phenotype involved is continuous. In particular, the pipeline is more efficient for investigating small sets of genotyped SNPs defined in high dimensional spaces that may be associated with continuous phenotypes, rather than for the investigation of whole genome variants. The pipeline, which employs a consensus approach based on the random forest, was able to rapidly identify previously unseen key SNPs, that are significantly associated with the platelet response phenotype, which was used as our complex trait case study. Several of these SNPs, such as rs6141803 of *COMMD7* and rs41316468 in *PKT2B*, have independently confirmed associations with cardiovascular diseases (CVDs) according to other unrelated studies, suggesting that our pipeline is robust in identifying key genetic variants. Our new pipeline provides an important step towards addressing the problem of ‘missing heritability’ through enhanced detection of key genetic variants (SNPs) that are associated with continuous complex traits/disease phenotypes.

## Introduction

Genetic association studies (GASs) allow scientists to study and analyse SNPs associated with complex traits or diseases. The traditional approach for genetic association (GA) analysis is to analyse one SNP at a time. However, multiple SNP analysis approaches have recently received much attention, and different strategies have been designed and adopted [1]. For instance, the widely used standard multiple SNP analysis approach is the forward stepwise method [2]. Other approaches include variants of penalised regression methods [3,4] and a compendium of the burden tests methods for analysing and detecting rare variants [5–9]. Besides these improvements, approaches that are computational and bioinformatics-based, are likely to complement the biostatistical methods and further improve crucial SNPs identification, and hence, further addressing missing heritability [10–12]. Here, we describe a novel, integrated, heuristic-based, hybrid analytical computational pipeline, for rapidly detecting novel or key genetic variants that are associated with complex traits continuous phenotype. The pipeline combines the power of random forests (RF) [13] and regularised regression methods, using ridge and least absolute shrinkage and selection operator (lasso) [14,15] for the analysis of SNPs in GASs, in addition to the stepwise method. The pipeline has been also coupled with a feature selection method known as Boruta [16] for further improving the key SNPs identification. In brief, this pipeline describes a consensus model based on the RF for identifying key genetic variants (SNPs) for further biological interpretation or predictive purposes.

This pipeline is able to select key SNPs associated with continuous phenotypic responses, and has been applied to analyse the effect of multiple SNPs and loci associated with platelet responses. The pipeline has identified several novel genetic variants significantly associated with platelet responses that were previously unidentified when only the standard stepwise method was used [17], yet it is also generally applicable for studying other continuous phenotypes.

Platelets are small anucleate cells packed with complex signalling machinery that enables them to react rapidly to damage in a blood vessel to prevent blood loss. During platelet functioning, several molecules (agonists) are involved in activating platelets, leading to platelet aggregation and thrombus formation [18,19], and culminates in the formation of a plug on the damaged blood vessel, which has been associated with CVD pathophysiology [20,21]. The platelet response to agonists is genetically regulated and highly variable among individuals, and over recent decades considerable success has been achieved in illuminating the genetic determinants that underpin platelet responses [17,22–24]. Despite this success, further understanding of the unaccounted genetic variability of the SNPs associated with platelet responses is required [25].

Using our approach, we analysed the genotyped SNPs data obtained from a previous functional genomic study that focused on understanding the genetic association underlying the platelet responses to agonists [17]. The analytical method deployed in the previous study was based on the forward stepwise method, which is argued to be statistically sub-optimal [26] and tends to omit key genetic variants, particularly those with strong linkage disequilibrium [27]. In the previous study, four platelet responses were involved: 1. P-selectin exposure (a marker of degranulation) in response to adenosine diphosphate (ADP) agonist (denoted by PA), 2. Fibrinogen binding in response to ADP (FA), 3. P-selectin in response to the GPVI specific agonist cross-linked collagen-related peptide (CRP-XL) (PC), and 4. Fibrinogen binding in response to CRP-XL (FC).

Here, we critically evaluate our new approach against the previous method using the same data [28]. Furthermore, we show that using our pipeline enhances our ability to identify key significant SNPs that are associated with platelet responses while also assessing the confidence level. Additionally, we tested the pipeline with the age covariate and we demonstrate that it has promising potential in accounting for further heritability of platelet responses and other continuous phenotypes.

## Materials and methods

The genetic association (GA) data were acquired from the bloodomics project [28], comprising 1430 SNPs (chosen from genomic regions in the vicinity or within candidate genes involved in the platelet responses or signalling pathways) for 462 individuals with descriptions of their effects on four platelet signalling pathways or platelet responses (i.e. PA, PC, FA, and FC). The data are therefore highly dimensional with the number of all SNPs ‘p’ greater than the number of observations ‘n’ (i.e. p > n). The platelet responses of our interest are quantitative and are normally distributed continuous trait phenotypes with n(1,0). These were measured in a previous study by flow cytometry [28], through the expression level of the two released molecules, i.e. fibrinogen (F) and P-selectin (P), after the platelet has been activated by agonists ADP (A) and CRP-XL (C). The SNPs’ genotypes were represented using the dummy variables 1, 2, and 3 corresponding to major homozygous, heterozygous, and minor homozygous respectively.

### The computational pipeline

The use of RF as an efficient tool for dealing with high dimensional data in the biomedical and life science has been elucidated in a previous review [23]. Our approach is a two staged analysis involving RF based on the work of Schwarz et al. [24], which is a standard for SNP discovery, as further explained by Goldstein et al. [25]. The detailed description of the pipeline is given below.

Using this dataset, we iteratively trained the random forests (RF) models, which were used to select the useful k SNPs from p. In this case, each iteration based on the *ntree* (the number of trees used in generating RF model), an RF regression model was trained for each of the four platelet responses in the dataset using all p SNPs. Then, the top 40 (k) among the overall ranked SNPs were selected using the permutation variable importance (VI) feature score measure [13]. We used an approximation of $\sqrt{p}$ as a cut-off value for selecting the top ranked k SNPs in each of the four platelet responses. The k SNPs were used as a baseline for further selecting key significant SNPs in the pipeline.

For each iteration, the RF model was retrained using the k SNPs to examine whether the model has improved. The performance improvement was observed with the increase in the value of *ntree*, starting from 500 up to 3000 trees (i.e. 500, 1000, 2000, 3000 for iterations 1, 2, 3, and 4 respectively) where the models exhibited a stable performance. The relative increase of *ntree* was shown to significantly increase the performance, and proven to enhance the selection of the relevant variables [29]. The performance of the RF models was evaluated using Eq 1.

$$R^{2}=1-\frac{\sum {\left(P_{observed}-P_{predicted}\right)}^{2}}{\sum {\left(P_{observed}-{\bar{P}}_{observed}\right)}^{2}}$$ (1)

where:

*R*^2^ is the root mean squared, *P*_*observed*_ and *P*_*predicted*_ are observed and predicted platelet responses respectively for each of the FA, PA, FC, and PC. ${\bar{P}}_{observed}$ is the mean platelet responses for each of the FA, PA, FC, and PC.

For each iteration, the k SNPs were further passed through the designed layer of an ensemble of (regularised) regression methods, which were used to find highly significant SNPs associated with platelet responses. Our rationale for devising this layer was to potentially increase the likelihood of identifying many significant SNPs based on the varying performances of the individual methods [30]. An additional aim in applying this layer was to increase the power of detecting significant SNPs that are likely to be missed by any of the other methods.

In our implementation of this layer, we used ridge and lasso, in additional to the stepwise forward methods. The stepwise forward method was initially used to examine the number of SNPs that would have been selected relative to the previous study [17] using the same data. We included lasso to retain potentially sparse interactions among the genetic variants [31]. Ridge regression was applied to take into account potential multicollinearity among SNPs, particularly those with strong linkage disequilibrium (LD) [32].

We collated and tested the SNPs resulting from each model generated from the different selected regression methods to find those that were significantly associated with FA, FC, PA, and PC platelet responses. The significant SNPs from each method were parametrically tested and selected based on the cut-off p-value of < = 0.01.

Table 1 demonstrates the effect of relatively increasing the RF’s *ntree* parameter on the variance of k SNPs and significance of regression models.

**Table 1:** The evaluation performance of the models in the pipeline.

Random Forests (RF) Run				RF+Stepwise	RF+Ridge regression	RF+Lasso
#Iterations	Number of trees (*ntree*)	% Variance all (p) SNPs	% Variance k SNPs	Model significance (r-squared & p-values)	Model significance (%Variance)	Model significance (r-squared & p-values)
1	500	-0.67	8.58	0.088 & 4.965e-09	14.7	0.096 & 1.83e-09
2	1000	0.43	13.7	0.11 & 4.771e-11	17.83	0.10 & 8.1e-10
3	2000	0.23	16.66	0.111 & 5.4e-11	18.77	0.138 & 7.108e-13
4	3000	0.51	17.94	0.13 & 1.49e-12	17.5	0.16 & 6.61e-15

For each iteration, the RF model performance was evaluated based on the increase of % variance when the model was run using p SNPs and selected k important SNPs. This was further followed up by passing the k SNPs into the multiple regression methods, where the confidence of each model was subsequently evaluated and its significant SNPs were examined. This was repeated until the models showed convergence. Note, the % variance is a negative number (-0.67) in the first iteration, i.e. ntree = 500. The negative number indicates that the prediction is very poor due to incorporating all SNPs (p) in the full model, a situation where many bad variables (SNPs) might be included [33,34].

Thus, from Table 1, the increase in the variance explained by the RF, and confidence of the intermediate regression models might be an indicative feature of the importance of the selected k and highly significant SNPs respectively. A similar pattern is observed when the pipeline is validated using the simulated data (see the section titled ‘Validation of the pipeline’). This further supports the work of Paul et al. [35] and Strobl et al. [29] who showed that the variables selected using the VI measures are likely to be statistically significant, and the increase in the value of ntree plays a significant role in the selection of the relevant variables respectively. The k SNPs from the optimal or converged RF model (i.e. final iteration when the ntree = 3000) were thus used to find the most significant and key SNPs in the final consensus approach.

Furthermore, for each iteration, the k SNPs were alternatively passed through Boruta method [16] layer, which is an RF-based method normally used to select all relevant important features. The Boruta method has previously shown the relative robustness in selecting potentially important genes [36]. In our approach, the Boruta method was applied to enhance the consensus identification of the most significant SNPs, by independently examining the significant SNPs relative to those selected by the regression methods layer, in addition to identifying new ones. The Boruta method finds k′ relevant (important) SNPs from k. The significance (or the importance) of SNPs in the Boruta method is measured using the Z-score.

**Fig 1:**
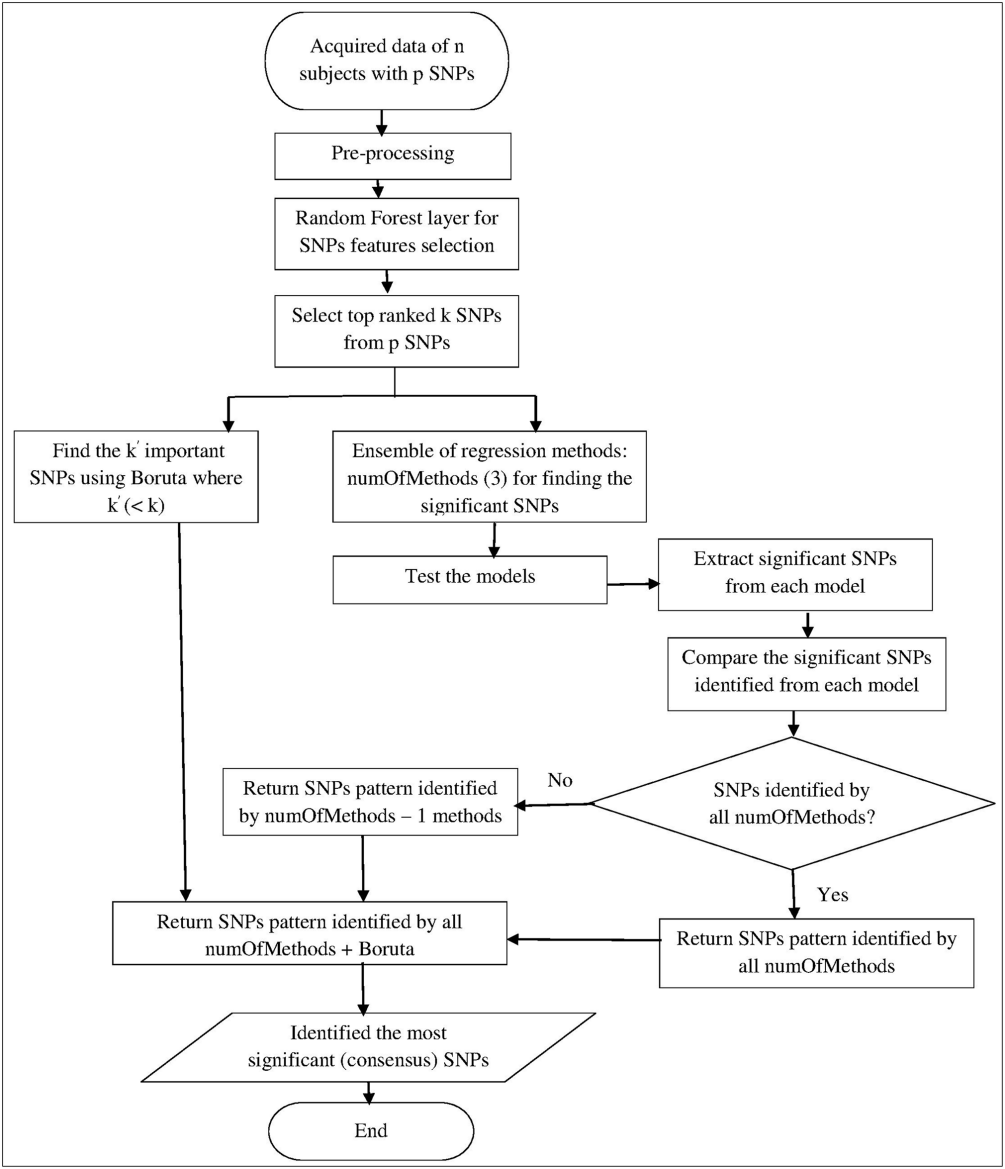
Flowchart showing the general methodological approach underpinning the pipeline. In high dimensional genetic data of n samples with p genotyped SNPs, the number of SNPs was first reduced from p to k by means of the RF layer. The selected k SNPs were further reduced by means of two alternative methods, the ensemble of three regression methods and the Boruta method. The most significant SNPs (key SNPs) are those that were selected by majority of the methods, i.e. in consensus, during the final iteration.

Once we had determined the optimal state for the pipeline (i.e. in the final iteration where ntree = 3000), we extracted and compared the SNPs from each method in the regression layer and the Boruta method to discover which of those were found to be the most significant by consensus, thereby confidently identifying them to be key genetic variants (Fig 1).

Based on the examined significant SNPs in different intermediate models in each iteration (i.e. ntree) of the pipeline, a confidence level was then assigned during the final iteration to verify that the selected key significant SNPs were not false positives. The confidence level also allowed us to ensure that true key significant SNPs (true positives) were not rejected, due to either being selected by a single method or being completely missed out in the final iteration when the pipeline converges. In order to assign a confidence score, a plot was created showing the frequency of the selected significant SNPs in the intermediate models in every iteration (see the Results and discussion section). The higher the frequency of appearance in the intermediate models, the greater the confidence score, or higher likelihood of being true key significant SNPs (true positives), i.e. during the observed convergence, if the same SNP appears in different intermediate models and in several iterations, then it is more likely to be a true positive. The confidence level of the selected significant SNP was then determined by taking the ratio of the frequency of appearance of a SNP (p_m_) in the intermediate models in all iterations (i.e. ntree = 500, ntree = 1000, ntree = 2000, and ntree = 3000) to the normalised total number of the models multiplied by total number of iterations. (Eq 2)

$$Confidence\;for\;SNP\;\left(P_{m}\right)=\;\frac{frequency\;of\;SNP\;\left(P_{m}\right)\;in\;the\;models\;in\;all\;iterations}{total\;number\;of\;models\;\times \;total\;number\;of\;iterations}$$ (2)

From Eq 2, a minimum threshold confidence level can be set, for instance, any score greater than 0.5 is more likely to be a true positive significant SNP.

### Initial implementation of the pipeline

#### Random forest

We used the randomForest [37] package in the R language [38] to run the RF layer.

#### Regression methods

The regression methods that were applied in the pipeline, are based on the standard linear regression model given by Eq 3:

Y=Xβ+ ε (3)

where:

*Y* is the response phenotype of concern (i.e. platelet responses PA, FA, PC, and FC for individuals), which is (*n x 1*) vector of dependent variables; *X* is a (*n x p*) design matrix, in this case are the SNPs genotype-coded with 1 for major homozygous, 2 for heterozygous, and 3 for minor homozygous; *β* is a (*p* x 1) vector of regression coefficients *β*_*j*_, *j* = (1,…,*p*); and *ε* is an assumed vector of normally distributed random errors with mean 0 and variance (*σ*^2^). So our model is a relationship between the continuous phenotype Y (i.e. platelet responses) determined by weighted SNPs X_*p*_ of *n* individuals.

#### Stepwise forward regression

We applied the stepwise regression with forward selection method, after filtering the SNPs using RF. Generally, the forward stepwise selection method starts with a null model and allows one SNP at a time to enter the model, based on which SNP is most correlated with each of the platelet responses, i.e. the addition of the SNP in the model depends on the SNP that gives the highest significant improvement in fit [2]. The selected SNPs in the stepwise model were tested for significance using the Wald test. We implemented the stepwise regression using package LEAPS [39] in R.

#### Shrinkage methods

Shrinkage methods [30] use a regularisation strategy to further penalise SNPs from k SNPs from the RF layer, assuming that the underlying RF functioning might possibly select SNPs that are not significant. This further simplified and enhanced the selection of highly significant SNPs. We applied the shrinkage methods using the ridge regression and lasso with R packages ‘ridge’ [40] and ‘glmnet’ [41] respectively. In applying the glmnet package, the family option is set to “gaussian” as the response phenotypes (platelet responses) are quantitative and assumed to follow the Gaussian distribution.

(1) Ridge Regression (RR) Model

Based on the model given by Eq 3 above, we sought estimates of regression coefficients that would determine the SNPs with higher effects to our phenotype (*Y*). The coefficients could be determined using the ordinary least square method (OLS), which is the standard approach and is given by Eq 4.

$$\hat{\beta }=\;{\left(X^{\prime }X\right)}^{-1}XY$$ (4)

However, this equation does not work particularly in the context of genetic data where collinearity is common among SNPs due to the high LD [27]. The ridge regression [14] was applied to ensure that potential collinear SNPs were kept in the models, particularly those in the strong LD. RR shrinks regression parameters by penalising their size and reducing towards zero using the computed ridge shrinkage parameter (lambda). The optimal shrinkage parameter helps to identify the regions where the model parameters are stable and controls the classical trade-off between the high bias and variances, which commonly occur when there are large number of parameters and collinearity among SNPs. Thus, Eq 5 shows the RR model for estimating the regression coefficient.

$$\hat{\beta }=\;{\left(X^{\prime }X+\;\lambda I\right)}^{-1}XY$$ (5)

where, the lambda (*λ*) is a ridge parameter, which determines the degree of shrinkage. I is a *p x p* identity matrix. Adding the term *λI* in the model, reduces the coefficient estimates towards each other, potential collinearity among SNPs, and eliminates the possibility of matrix *X*′*X* being singular. The parameter *λ* is selected between 0 and ∞ values. If λ = 0 then the RR model is turned to be ordinary least square (OLS) solution, and if λ = ∞ then the model would behave as if no parameters have been estimated, and the solution would be the mean of the response variable, i.e. $\bar{Y}=\beta_{0}$.

We used an automatic lambda selection method for an optimal lambda selection [42], and the Wald test for testing the significant SNPs from the RR.

(2) Lasso

We also applied lasso [15] to the selected k SNPs from the RF model to possibly retain sparse interactions among the SNPs [31]. The lasso model is given by:

$${\hat{\beta }}^{lasso}=\begin{array}{c} argmin\\ \beta \end{array}\sum_{i=1}^{N}{\left(y_{i}-\beta_{0}-\sum_{j=1}^{p}x_{ij}\beta_{j}\right)}^{2}+\;\lambda \sum_{j=1}^{p}\left|\beta_{j}\right|$$ (6)

where:

y_i_ is the vector of particular response phenotype (among PA, FA, FC, or PC) for observation i; X is a design matrix of SNPs and ${\hat{\beta }}^{lasso}$ are the lasso coefficient estimates of the SNPs; the lambda term is the weight given for the regularisation term (L1 norm), which sparsely picks the SNPs entering the model, when the tuning parameter is very small or exactly zero.

The SNPs coefficients from the lasso models were extracted based on the selection of the smallest optimal lambda (or tuning parameter) value using 10-fold cross validation [43]. The lasso models generated the sparse matrix of SNPs coefficients estimates. The SNPs with relative large coefficient estimates from the sparse matrix were selected and tested in a stepwise manner using the partial F-test [44] to determine the individual SNP’s significance level in the model.

#### Boruta method

Boruta is an all-relevant feature selection method, which provides an improved mechanism for selecting an important feature or variable from the RF using a Z-score. It is a wrapper algorithm, which ranks the features from the RF through an improved Z-score. The applied Z-score within Boruta provides the statistical significance, and hence the relevance of the selected important variable or feature [16]. The Boruta was used with the aim to add more weight to the consensus selection of the key SNPs in addition to the regression layer.

Boruta was run using Boruta package in R [16]. In running Boruta, the maximum number of iterations (maxRun) was set to 100.

### The performance of the pipeline with the inclusion of covariates

The pipeline is specifically designed for analysing predetermined, genotyped SNPs to identify the most significant SNPs (key SNPs) that are associated with continuous complex trait phenotypes and would have been likely to be missed by other approaches such as stepwise. The pipeline was initially applied to alternatively analyse the combined effect of the SNPs and benchmarks the results against those obtained from the stepwise forward approach [17], which did not need to take into account the covariates, such as age, gender, height, weight, ethnicity, aspirin taking, medication, smoker, contraceptive pill, because they were already treated separately during the data pre-processing stage of the Bloodomics project [28].

Nevertheless, we have re-tested our pipeline to demonstrate the incorporation of an example key covariate for CVD: age. The approaches for handling covariates in determining the effect of SNPs on the phenotype using RF have been well elucidated by Nonyane and Foulkes [45]. In running the pipeline, the age was included as a numeric type and potential predictor together with SNPs under the additive model.

Table 2 shows the performance of the RF models when the pipeline is run with age as a covariate in identifying the most significant SNPs associated with PA platelet response.

**Table 2:** The performance of the RF with and without age as a covariate in determining the PA platelet response.

Random Forests (RF)–SNPs *without* age incorporated as a covariate				RF—SNPs *with* age incorporated as a covariate	
#Iterations	Number of trees (*ntree*)	% Variance all (p) SNPs	% Variance k SNPs	% Variance all (p) SNPs	% Variance k SNPs
1	500	-0.16	*11*.*85*	0.17	*13*.*95*
2	1000	-0.5	*14*.*29*	-0.69	*14*.*46*
3	2000	-0.06	*16*.*86*	0.15	*18*.*54*
4	3000	0.33	*15*.*92*	0.12	*16*.*36*

From Table 2, there are an observed marginal increases in the variation explained by the RF models when age is included as a covariate. The residuals plots are shown in the S1 and S2 Figs. The significance of the regresion models due to the covariate in the intermediate regression models are shown in the S6 Table. Few intermediate models have higher significance in the early iterations when age is included as a covariate comparing than when it is excluded.

### Validation of the pipeline

To validate the pipeline, we randomly simulated 460 subjects containing 1400 artificially genotyped SNPs with their associated continuous phenotype (See S2 Text, for R code to reproduce the data). The simulated phenotype is a univariate normal distribution with n(0,1). The genotypes of these artificial SNPs follow the standard representation consisting of 1, 2, and 3, which represents major homozygous, heterozygous and minor homozygous respectively. This simulated data set was applied to the pipeline. The RF and the multiple regression models using k SNPs were observed to improve as *ntree* was increased in each iteration starting from 500, 1000, 2000, until 3000 trees, where the variance and confidence of the models started to converge (Table 3).

**Table 3:** The evaluation performance of the pipeline for the simulated SNPs.

Random Forests (RF) Run				RF+Stepwise	RF+Ridge regression	RF+Lasso
#Iterations	Number of trees (*ntree*)	% Variance all (p) SNPs	% Variance k SNPs	Model significance (r-squared & p-values)	Model significance (%Variance)	Model significance (r-squared & p-values)
1	500	1.14	11.84	0.12 & 7.342e-12	24.36	0.14 & 2.482e-13
2	1000	1.71	16.36	0.14 & 1.042e-13	27.51	0.20 & 2.2e-16
3	2000	2.55	21.31	0.15 & 1.082e-14	28.97	0.22 & 2.2e-16
4	3000	1.6	19.34	0.13 & 1.604e-12	28.2	0.19 & 2.2e-16

The models’ patterns observed using the artificial SNP data are shown to reflect those observed with the real SNP data (Table 1), even though the convergence in this case, seems to be in the third iteration when the *ntree* was 2000.

## Results and discussion

Firstly, our new approach has identified several significant SNPs that are associated with all platelet responses and are consistent with the previous study [17], and Tables A and B in S1 File. Importantly, we have also discovered numerous additional SNPs that are significantly associated with platelet responses and were not previously identified, or previously found to be insignificantly associated with platelet responses using the forward stepwise method. Tables 4 and 5, and Tables in S1 and S2 Tables, show the overall significant and key SNPs identified by our pipeline and the previous method that are associated with PA, FA, FC, and PC platelet responses respectively. From the results, we can establish a consensus approach for the identification of key SNPs, which are those identified as significant by the three out of four approaches within the pipeline.

**Table 4:** Consensus identification of the most significant SNPs associated with PA platelet response.

		Stepwise (Jones et al 2009)	RF with Stepwise	RF with Ridge regression	RF with LASSO	RF with Boruta (P = 0.01)	Consensus (3/4)
Platelet response type		PA	PA	PA	PA	PA	PA
SNPs ID	Gene/Location						
rs17229705	*VAV3*	✔ (0.0009)	×	×	×	×	
rs3788337	*GNAZ*	✔ (0.0009)	×	×	×	×	
rs5227	*PTGS2*	✔ (0.01)	×	×	×	×	
rs1778614	*ITPR1*	✔ (0.003)	×	×	×	×	
rs246406	*ITGA2*	✔ (0.002)	×	×	×	×	
rs11631474	*MAP2K5*	✔ (0.007)	×	×	×	×	
rs851007	*MAPK14*	✔ (0.003)	×	×	×	×	
rs6141803	*COMMD7*	×	✔ (0.0033)	×	✔ (0.0006)	✔	✔
rs6442896	*ITPR1*	× (0.049)	✔ (0.0006)	✔(0.0002)	✔(0.0021)	✔	✔
rs3730051	*AKT2*	× (0.031)	✔ (0.0002)	✔ (0.0031)	✔ (0.0002)	×	✔
rs1527480	*CD36*	× (0.449)	✔ (0.0021)	✔(0.0008)	✔ (0.0036)	✔	✔
rs8033381	*CSK*	× (0.792)	✔ (0.0018)	✔ (0.0082)	✔ (0.0038)	×	✔
rs10061730	*ITGA2*	× (0.517)	×	×	✔ (0.0005)	×	
rs2292867	*ITGB3*	×(0.039)	×	✔ (0.017)	✔ (0.0080)	×	
rs2300065	*SKP1*		×	✔(0.0138)	✔(0.0164)	×	
rs3212391	*ITGA2*	×	✔ (0.0002)	×	×	✔	
rs6433658	*ITPR1*	×	×	×	×	✔	
rs6442895	*ITPR1*	×(0.029)	×	×	×	✔	
rs17041401	*ITPR1*	✔(0.003)	×	×	×	✔	
rs3212386	*ITGA2*	×(0.378)	×	×	×	✔	
rs33443	*ITGA2*	×(0.547)	×	×	×	✔	
rs26682	*ITGA2*	×(0.126)	×	×	×	✔	
rs3212418	*ITGA2*	✔(0.013)	×	×	×	✔	
rs11742558	*ITGA2*	×(0.713)	×	×	×	✔	
rs7568033	*NFE2L2*	×	×	×	×	✔	

We select the consensus SNP if it has been identified by at least three methods, which means it has higher significance and hence is more likely to be a key genetic variant.

× indicates either the SNP was not identified by the method or previously identified as insignificant

✔ indicates the SNP was identified by the method.

Numbers inside the brackets after ✔ or × indicate p values of the SNPs calculated using Wald test, or partial F-test.

**Table 5:** Consensus identification of the most significant SNPs associated with FA platelet response.

		Stepwise (Jones et al 2009)	RF with Stepwise	RF with Ridge regression	RF with LASSO	RF with Boruta (P = 0.01)	Consensus (3/4)
Platelet response type		FA	FA	FA	FA	FA	FA
SNPs ID	Gene/Location						
rs11637556	*MAP2K1*	✔ (0.005)	✔ (0.0007)	✔ (0.0083)	✔ (0.0008)	✔	✔
rs10429491	*JAK2*	✔ (0.0006)	×	×	×	×	
rs3729931	*RAF1*	✔ (0.0001)	×	×	×	×	
rs41305896	*ITGA2*	✔ (0.001)	×	×	×	×	
rs350916	*MAP2K2*	✔ (0.001)	×	×	×	×	
rs17786144	*ITPR1*	✔ (0.002)	×	×	×	×	
rs11264579	*PEAR1*	✔ (0.004)	×	×	×	×	
rs41304345	*MADD*	✔ (0.003)	×	×	×	×	
rs1388622	*P2RY12*	× (0.058)	✔ (0.0001)	×	×	✔	
rs2071676	*CA9*	×	✔ (0.0122)	✔(0.0058)	✔(0.0098)	✔	✔
rs1491978	*P2RY12*	× (0.092)	×	×	✔(0.0003)	✔	
rs1537593	*CD36*	× (0.731)	×	×	✔(0.0058)	✔	
rs9895150	*ITGB3*	× (0.177)	×	✔(0.0193)	✔(0.0141)	×	
rs1038639	*ITPR1*	× (0.138)	×	✔(0.0019)	✔(0.0006)	✔	✔
rs10499858	*CD36*	× (0.129)	✔ (0.0012)	×	×	✔	
rs7034539	*JAK2*	× (0.061)	✔ (0.0053)	✔(0.0058)	✔(0.0077)	×	✔
rs3742633	*PRKCH*	× (0.985)	✔ (0.0172)	×	✔(0.0075)	×	
rs41282607	*MAPK1*	× (0.2)	✔(0.0113)	✔(0.0034)	✔(0.0087)	✔	✔
rs41305272	*MAP2K5*	× (0.955)	×	✔(0.0127)	✔(0.01)	✔	✔
rs7180408	*GTF2A2*	×	×	✔(0.0191)	×	×	
rs3736101	*MADD*	✔(0.015)	×	×	✔(0.0076)	×	
rs304076	*ITPR1*	× (0.395)	×	×	✔(0.0083)	×	
rs17204437	*P2Y12*	× (0.499)	×	×	✔(0.001)	✔	
rs6787801	*P2Y12*	× (0.448)	×	×	×	✔	
rs3173798	*CD36*	× (0.085)	×	×	×	✔	

We select the consensus SNP if it has been identified by at least three methods, which means it has higher significance and hence is more likely to be a key genetic variant.

× indicates either the SNP was not identified by the method or previously identified as insignificant.

✔ indicates the SNP was identified by the method.

Numbers inside the brackets after ✔ or × indicate p values of the SNPs calculated using Wald test, or partial F-test.

**Fig 2:**
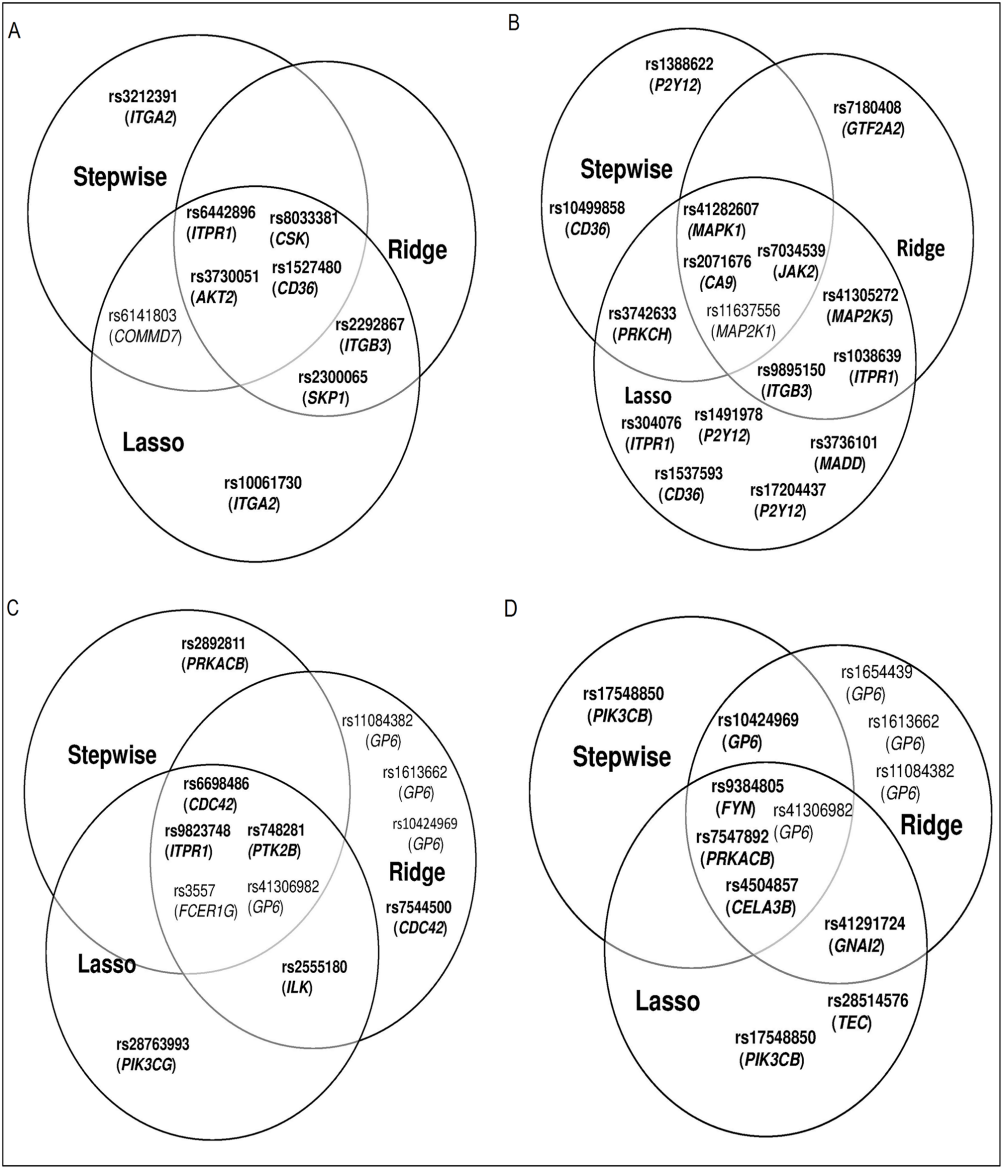
Venn diagrams for identifying significant and key SNPs associated with the all four platelet responses, which were identified by the regression layer in the pipeline. The identified significant SNPs that are associated with (A) PA (p-selectin in response to adensine diphsphate), (B) FA (fibrinogen binding in response to adenosine diphsphate), (C) FC (fibrinogen binding in response to collagen-related peptide), and (D) PC (p-selectin release in response to collagen-related peptide) platelet responses. The newly detected SNPs, or those reported as insignificant in the previous study are shown in bold. The key SNPs are found in the intersection regions and are detected by a consensus of the three methods.

In Fig 2A–2D we provide Venn diagrams of the overall significant and key SNPs identified by the multiple regression methods layer within the pipeline. These diagrams provide an alternative way of observing the key SNPs lying within the intersection regions.

**Fig 3:**
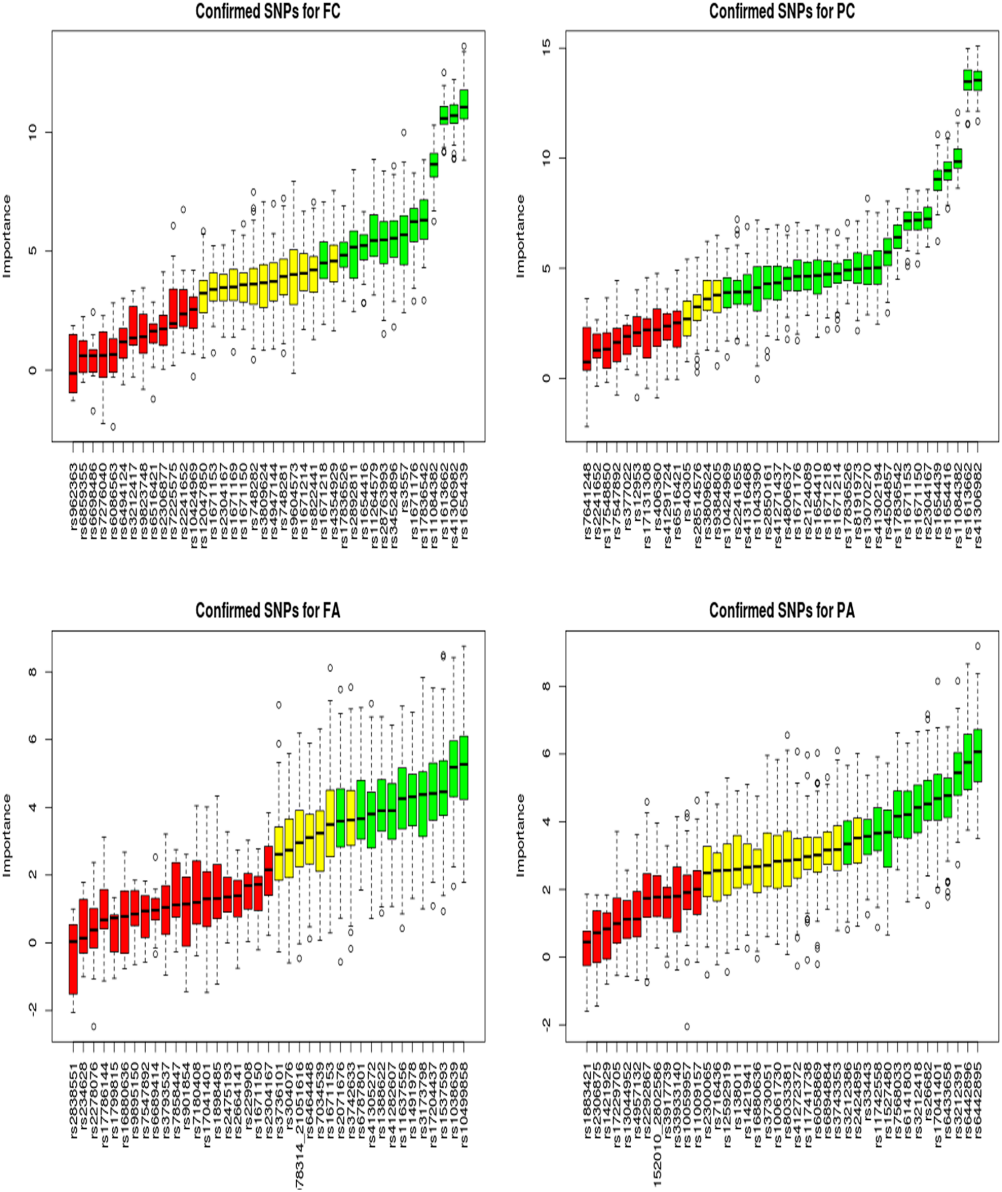
The Boruta method plot showing SNPs that are associated with four platelet responses. These are SNPs associated with (A) FC (fibrinogen binding in response to collagen-related peptide), (B) PC (p-selectin release in response to collagen-related peptide), (C) FA (fibrinogen binding in response to adensine diphsphate), and (D) PA (p-selectin in response to adensine diphsphate) platelet responses. The green, yellow and red boxplots are the confirmed important, tentative, and rejected SNPs respectively. The confirmed important SNPs are the significant SNPs associated with platelet responses. The selected significant SNPs here add more weight to the already identified SNPs from other methods, which may improve the consensus identification of the key SNPs and highlight other significant SNPs potentially missed by other methods in the pipeline.

Using the Boruta method [16] layer, we found that several of the identified significant SNPs that were associated with all four platelet responses, were also closely similar to those identified by the regression methods layer (Fig 3A–3D).

This further improved the consensus selection of the most significant SNPs associated with the platelet responses and strengthens our confidence in their association with each platelet response phenotype, which may strongly imply that further experimental investigation of these SNPs is warranted. Moreover, using Boruta as an additional layer in the pipeline further enhances the discovery of significantly associated SNPs that may be missed by other methods in the pipeline.

**Fig 4:**
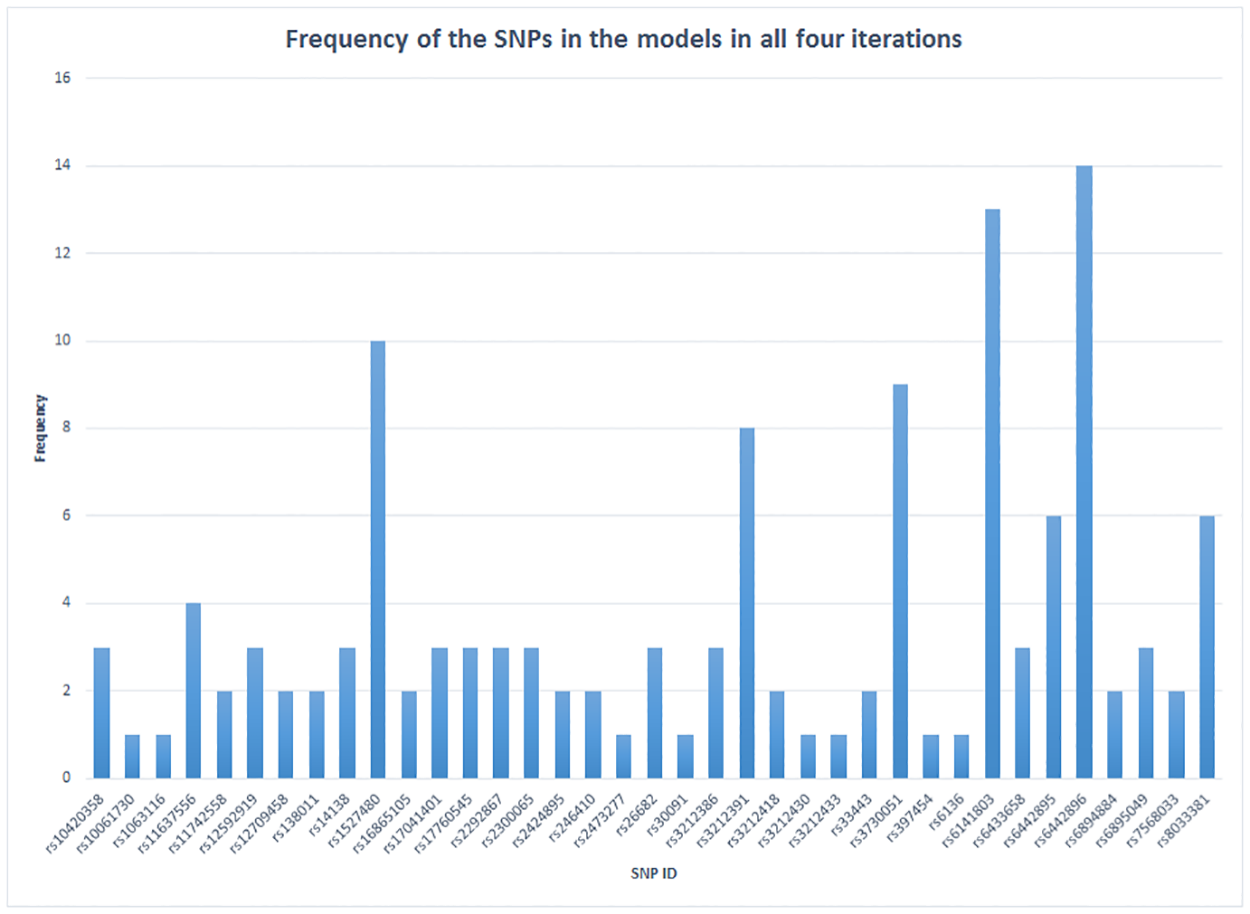
The frequency of the selected significant SNPs, which are associated with PA platelet responses in all iterations within the intermediate models. It can be seen clearly that some SNPs have relatively low or high frequencies, which mean they are more likely to be false or true positive key significant SNPs respectively. In our case, the maximum frequency is 16, which means the SNP appears in the four models in each of the four iterations.

For verifying the selected significant key SNPs in the final iteration are true positives, we applied the confidence level mechanism based on Eq 2 above. We initially visualise the identified significant SNPs in all iterations using the frequency plot. For instance, to assess the confidence of PA associated significant key SNPs, the plot showing the frequency of all significant SNPs in all iterations is initially created (Fig 4). S3 Table shows the frequency of each selected SNP in each iteration.

Therefore, from the data in Fig 4 and applying Eq 2, the SNP rs6141803 has appeared in the intermediate models 13 times in all iterations. The total number of models (methods) within the pipeline are 4. The total number of iterations are 4, i.e. four different RF run ntree sizes (ntree = 500, ntree = 1000, ntree = 2000, and ntree = 3000), thus, the confidence level of SNP would be 13/4*4 = 0.8125. This confidence score exceeds 0.5, and therefore, the selected SNP is more likely to be a true positive. Applying Eq 2 to data in Fig 4, and Table 3, we have identified with high confidence 7 key SNPs (rs1527480, rs3212391, rs3730051, rs6141803, rs6442896, rs6442895, and rs8033381) that are significantly associated with PA platelet responses.

**Fig 5:**
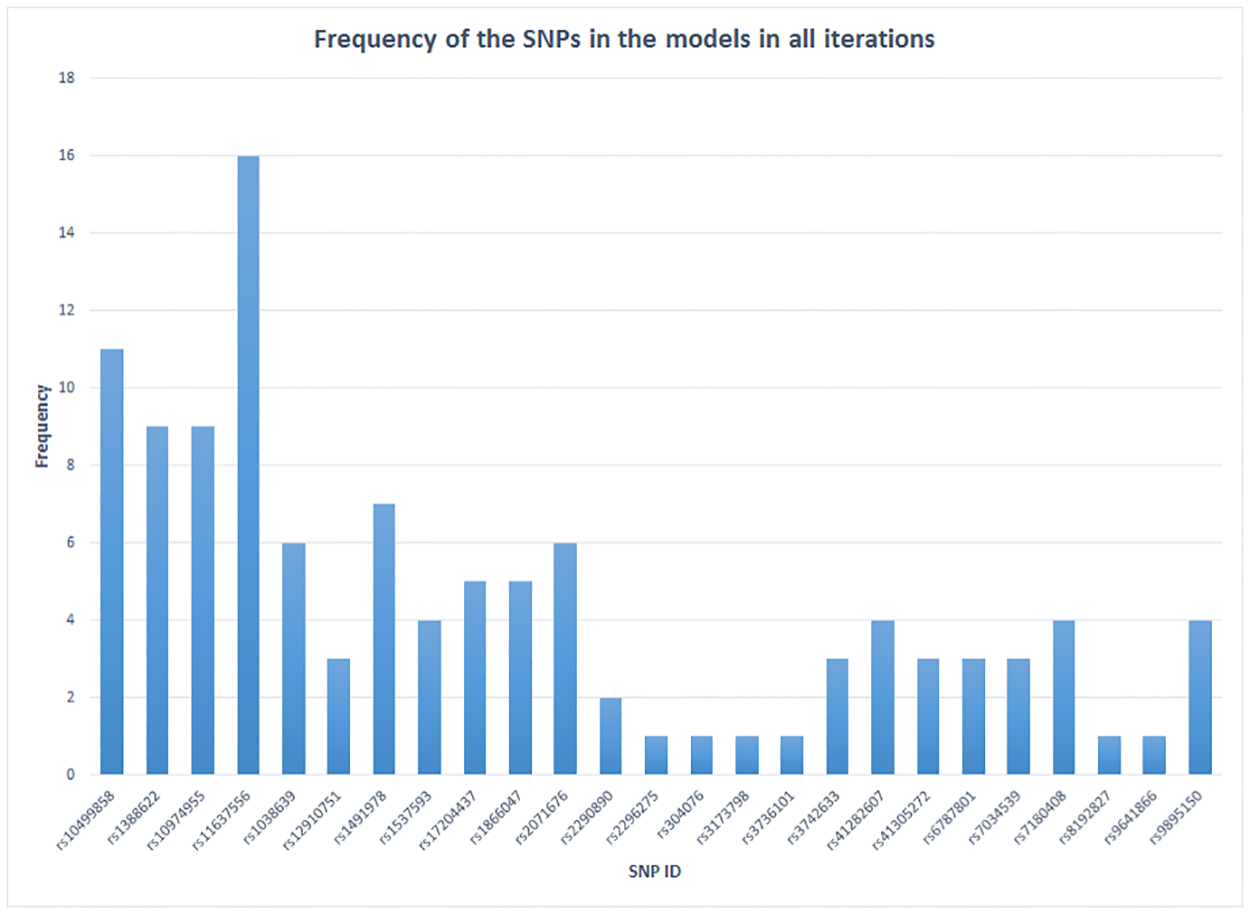
The frequency of the selected significant SNPs, which are associated with FA platelet response in all iterations within the intermediate models. A similar SNPs selection pattern as observed in Fig 4. Few SNPs are shown to be highly significant. For instance, rs11637556 in MAPK1 has been selected in each iteration.

Similarly, the FA platelet response associated SNPs are observed in the frequency plot in the Fig 5, prior to applying Eq 2 to determine the confidence scores. S4 Table shows the frequency of significant SNPs that were selected in every iteration.

From the data in Fig 5, the rs11637556 SNP *in MAPK1* has a confidence level of 16/4*4 = 1, (the highest confidence level for an FA platelet response associated SNP). Moreover. we have identified 7 key SNPs that are confidently associated with FA platelet responses (rs10499858, rs11637556, rs1388622, rs10974955, rs1038639, rs1491978, and rs2071676).

### Validation of the pipeline

**Fig 6:**
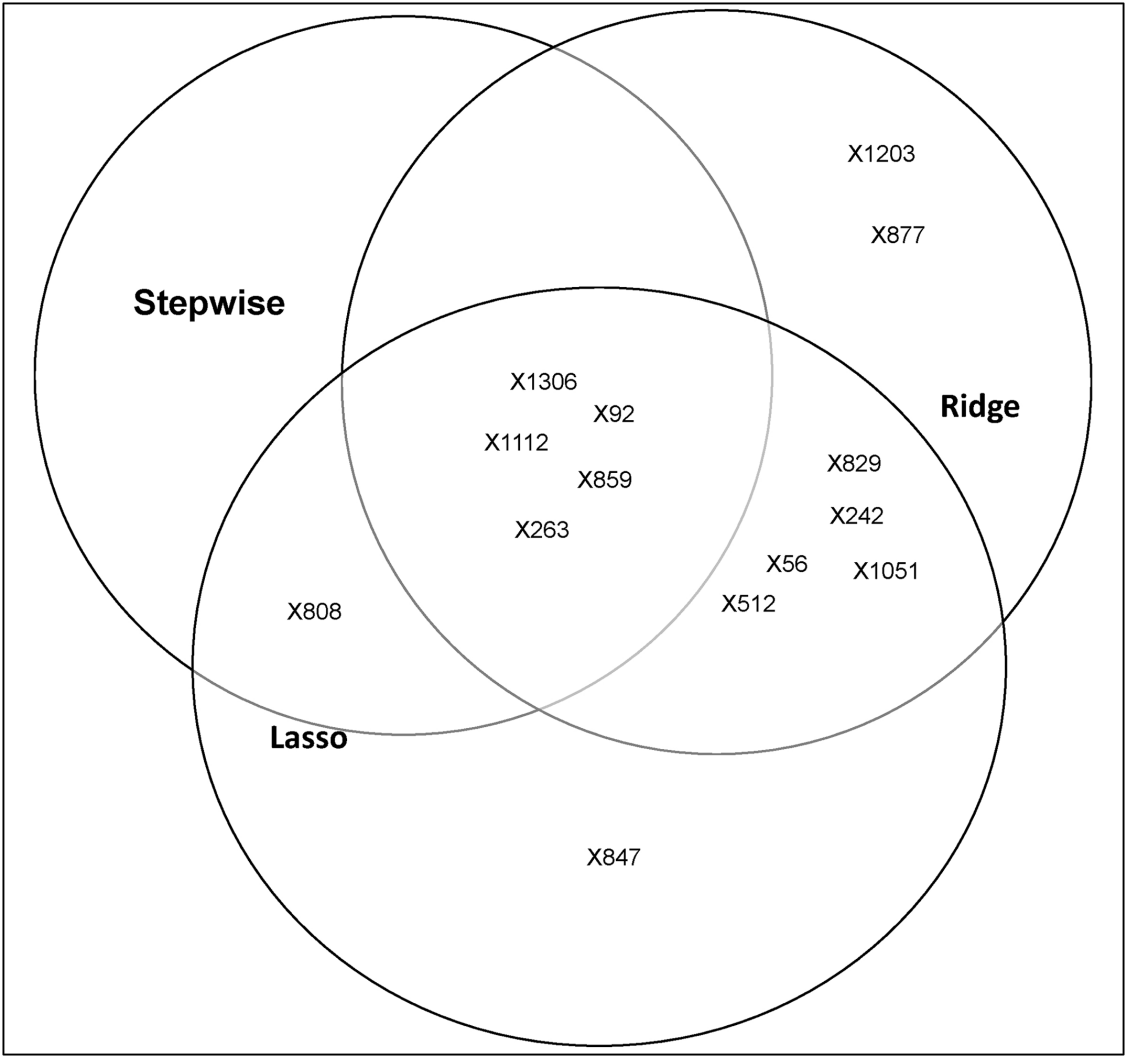
The visualisation of the selected key significant artificially simulated SNPs (intersection regions). Xm represents the identifier of the simulated genotyped SNP m. Several simulated SNPs were consistently identified to be significant by the multiple methods as occurred in the actual SNPs data set.

Several of the artificially simulated genotyped SNPs were identified consistently across the methods in the final iteration and were significantly associated with the simulated continuous phenotype. Fig 6 shows the visualisation of the artificially simulated key SNPs, which were identified by the regression based methods.

**Fig 7:**
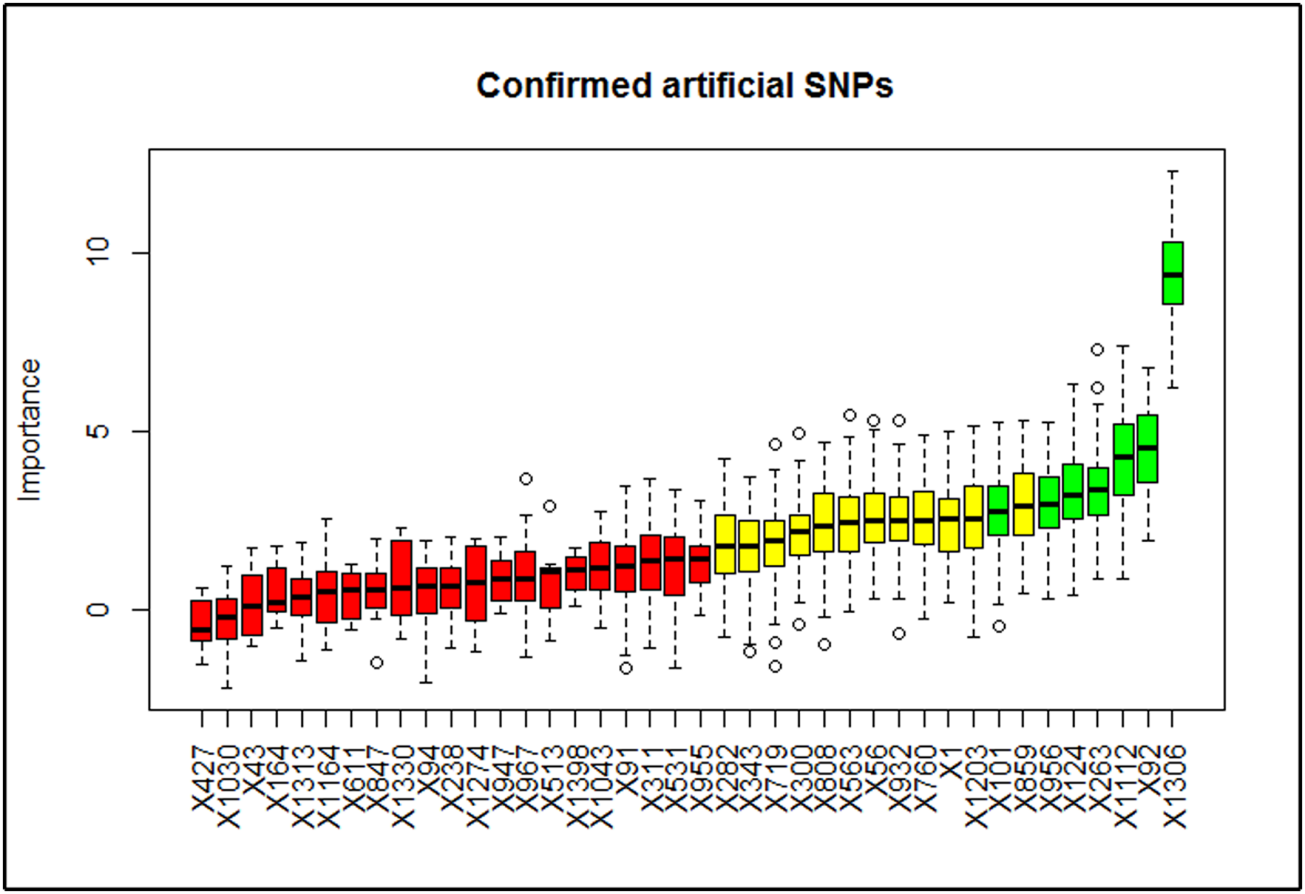
The confirmed selected artificially simulated key SNPs by the Boruta. Xm represents the identifier of the simulated genotyped SNP m. It can be seen clearly that most of the selected SNPs in the regression layer have been also selected by Boruta, which further enhanced key SNPs selection.

Furthermore, using the Boruta method, we identified several simulated artificial SNPs to be matched with those with key effect identfied in the regression based methods and thus, adding more weight to the consensus selection of the key SNPs. Fig 7 shows the Boruta plot for the selected artificial simulated SNPs.

Table 6 shows the selected key and significant artificial simulated genotyped SNPs in a consensus manner.

**Table 6:** The selected consensus artificial SNPs from the simulated data set.

Artificial SNPs ID	SNP’s significance in the models				
	RF with Stepwise	RF with Ridge regression	RF with LASSO	RF with Boruta (P = 0.01)	Consensus (3/4)
X1306	✔(4.15e-05)	✔(0.0003)	✔(1.73e-05)	✔	✔
X92	✔(0.0004)	✔(0.003)	✔(0.0014)	×	✔
X1112	✔(0.0017)	✔(0.00204)	✔(0.0013)	✔	✔
X808	✔(0.0013)	×	✔(0.0073)	×	
X859	✔(0.0034)	✔(0.0061)	✔(0.001)	×	✔
X263	✔(0.0021)	✔(0.0151)	✔(0.0061)	✔	✔
X829	×	✔(0.0065)	✔(0.009)	×	
X1203	×	✔(0.0171)	×	✔	
X242	×	✔(0.0075)	✔(0.003)	✔	✔
X56	×	✔(0.0135)	✔(0.0071)	✔	✔
X1051	×	✔(0.0122)	✔(0.005)	×	
X877	×	✔(0.0151)	×	×	
X512	×	✔(0.0019)	✔(0.0131)	×	
X847	×	×	✔(0.01)	×	
X760	×	×	×	✔	

Xm represents an identifier of the genotyped SNP m in the simulated data set. Several of the significant SNPs associated with phenotype were selected across the methods meaning that they are key significant SNPs associated with complex phenotype.

× indicates either the SNP was not identified by the method. ✔ indicates the SNP was identified by the method. Numbers inside the brackets after ✔ indicate p values of the SNPs calculated using Wald test, or partial F-test.

**Fig 8:**
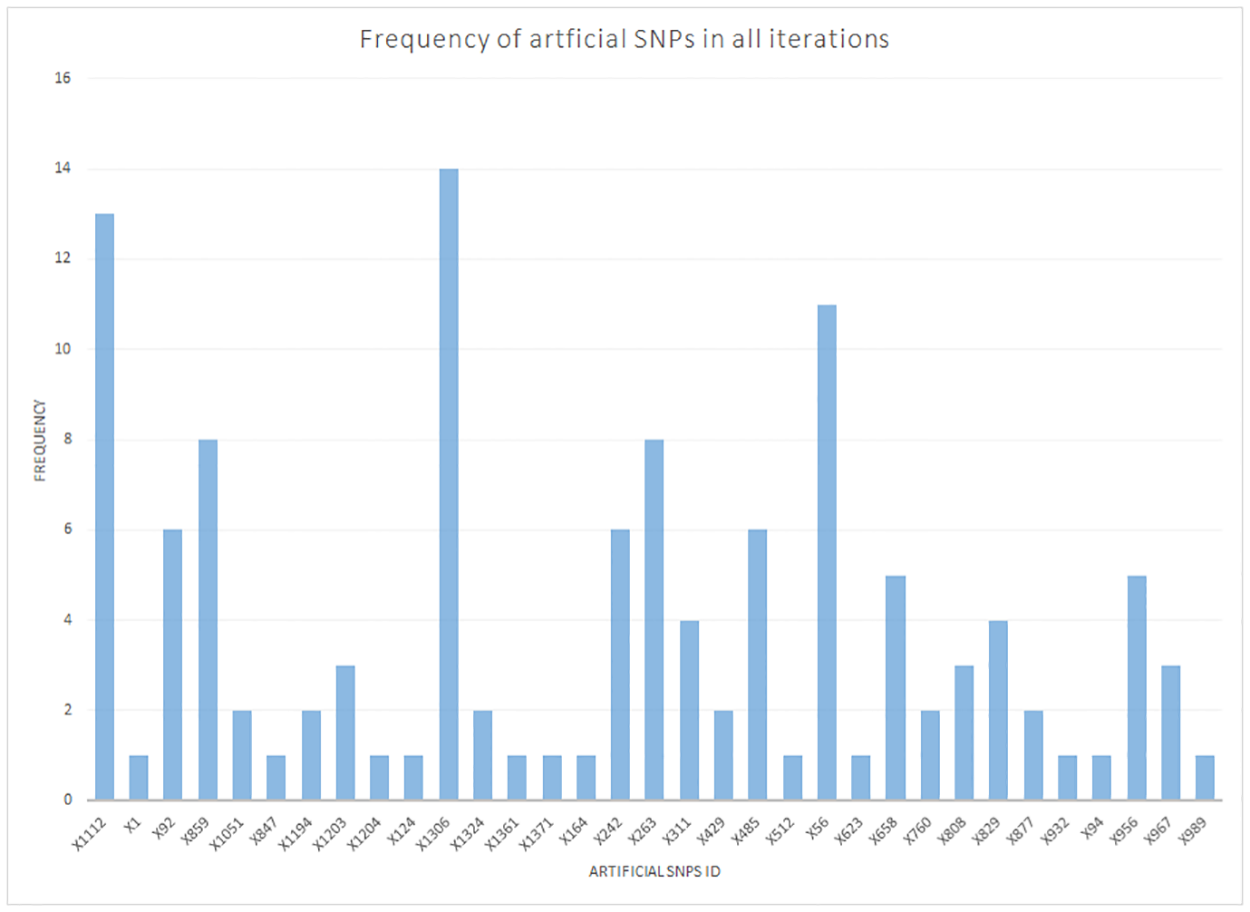
The frequency plot showing the overall selected significant artificially simulated SNPs in the intermediate models in all four iterations. The highly ‘enriched’ simulated SNPs can be easily identified.

For the identified significant simulated artificial SNPs, we plotted their frequency (Fig 8) and applied Eq 2 to determine true positive key SNPs. S5 Table shows the frequency of significant simulated artificial SNPs, which were selected in every iteration.

For instance, applying Eq 2 to the simulated significant SNP X56, the confidence level will be 0.6875, which has surpassed the minimum threshold confidence level and hence, is more likely to be a a true positive key SNP. In total we identified 8 artificially simulated SNPs that are confidently associated with the simulated phenotype.

Therefore, the similarity in the performance of the pipeline and its pattern of the results using both the real and simulated genotyped SNPs data sets, indicates that the pipeline is more likely to be robust when applied to other continuous phenotypes.

### Effects of age as an example covariate and the selection of key SNPs

We have found that in most cases, the key SNPs which were significantly identfied to be associated with the platelet responses when the pipeline is run age incorporated as a covariate are the same as those when age is not incorporated. For instance, for PA platelet response, most of the SNPs were identical to those selected when age is not included, signifying that the age might have a less significant effect when it is combined with SNPs in explaining the PA variation. S7 Table, shows the frequencies of the SNPs selection in the intermediate models associated with the PA platelet response in each iteration, when age is included as a covariate. S3 Fig, shows the plot, which illustrates the most frequent selected significant SNPs that are associated with PA for all iterations of the pipeline. All of the selected key SNPs are the same, except rs8033381, which was not selected under the presence of age as a covariate.

Furthermore, for the FA platelet response, nearly all the SNPs, which were identiftied to be significantly associated with FA when age is excluded are the same with those under the inclusion of age. However, in some stages of the pipeline run, age appears to have a likely association with FA platelet response, but in addition to other key SNPs. The plot in S4 Fig, with its related table in S8 Table, shows the different SNPs that are selected in every iteration;age is selected in the fourth iteration by the stepwise method with a p-value of 0.016.

We separately tested age with the key SNPs (rs11637556, rs1388622, and rs2071676) and found that it has a likely significance with FA (p-value = 0.05) along with rs1388622 and rs11637556 of *P2RY12* and *MAP2K1* respectively. Moreover, in almost every iteration of the RF, age was among the top ranked predictors, in addition to other SNPs, S5 Fig.

### Advantages to our approach

There are several advantages to our new combined approach. Firstly, the RF layer plays a crucial role in ensuring that potentially highly important SNPs are selected and passed through to the regression ensemble and Boruta layers. This use of the RF as an initial filtering stage is a well described standard approach for SNP discovery and plays a crucial role in selecting potentially highly important SNPs, using the appropriate *ntree* and VI parameters [29,46,47]. The selection bias introduced by the VI measure with the ranking approach has been shown to mainly affect predictors with different categories and scale of measurements [48], which is not the case in our study. In addition, the use of the VI measure with a ranking approach is still regarded as a useful strategy for selecting important SNPs for downstream analyses [49,50].

Secondly, it is possible to rapidly identify the key genetic variants, or markers, using a consensus of multiple alternate methods. Additionally, by introducing the multiple alternate methods layers, the likelihood of identifying other significant SNPs that might have been missed in one or more of the methods increases. This combination of methods in an integrated manner is a good approach for reducing false positives as multiple methods might be pointing to the same SNPs [51]. This potentially increases the chance of keeping functional SNPs associated with the phenotype, minimising the risk of ‘missing heritability’ [10], which is one of the thorny issues in GASs [11]. Moreover, based on this approach, the identified true complex trait associated key SNPs are more likely to be indicating the significantly overexpressed loci, which are likely to be proper candidates for follow-up experiments.

Furthermore, our pipeline is computationally adaptable and scalable to different implementations, particularly in the regression methods ensemble layer. It is possible to increase the number of (regularised) regression methods for optimising the detection of the key SNPs through consensus identification.

Furthermore, the computational speed of the pipeline means that is practical to implement as an additional tool. For the data set we used, the time taken to run the entire pipeline was 229.77012 secs on a modest quad core system running Ubuntu 14.04. Our pipeline does not necessarily aim to replace existing methods such as EMMAX [52] and PLINK [53], rather it may be used to supplement and further enhance the identification of key SNPs associated with continuous response phenotypes, with little additional computational overhead.

In addition, the pipeline may have an observed advantage over existing RF based methods in terms of its ability to identify other true trait associated SNPs. For instance, Boruta is the RF based method for relevant feature selection. We compared the SNPs that were obtained after running the pipeline with those from the Boruta method and found that the pipeline has the potential edge in identifying key SNPs, which might be missed by using only Boruta. For example, in the case of the PA associated significant SNPs, we found that the pipeline is able to identify rs3730051 in the *AKT2* locus as a key SNP, which was not recognised as a relevant important feature by the Boruta.

### Limitations/Caveats of the approach

The limitations of our approach are discussed below.

#### Sample size of the SNPs data

Furthermore, our pipeline is likely to be most suitable for genetic association studies with relatively small SNP datasets [54], and it appears to perform well when applied to the platelet responses data. However, this approach has not yet been tested or applied to genome-wide scale data e.g. with several million SNPs for association mapping. In such cases, the subspace SNPs selection methods could be initially employed [55,56], for selecting a subspace of informative SNPs and minimising the computational cost in generating trees, prior to using our approach.

#### Missing genotypes

Data with missing genotypes could be handled prior processing using different approaches [57–59]. For example, random imputation might be an option, which replaces the missing genotypes with the most frequent genotype based on the distribution of the SNPs genotypes (1, 2, or 3) across cases. For large numbers of missing genotypes, several established methods and tools, such as IMPUTE [60,61], Beagle [62] and PLINK could be used.

#### Long range LD and rare variants

The pipeline is solely generic in use for the identification of key significant SNPs within candidate genes associated with continuous phenotypic traits. For examining whether the identified SNPs are in long range LD [63], the pipeline could be supplemented with other methods or tools such as GLIDERS [64] and GWAS3D [65]. Furthermore, the pipeline has not been tested whether it is able to detect the rare variants. Instead, other approaches such as those proposed by Hoffmann et al. [8], sequence kernel association test (SKAT) [66], and kernel-based adaptive cluster (KBAC) [9] might be used accordingly for detection of rare variants.

### The newly identified SNPs and their biological and clinical significance

Our approach was able to discover numerous and previously undetected SNPs, which are significantly associated with the platelet response phenotype. Several of these SNPs have also been highlighted in other independent studies as being implicated in CVDs. The following examples underpin our results and serve to further strengthen our confidence in the ability of our approach to identify key genetic variants.

For example, the identified intergenic SNP rs6141803 in *COMMD7*, which is associated with PA was also identified in another platelet functional study [22] to be a likely risk factor for myocardial infarction. In addition, two *P2Y12* SNPs rs1491978 and rs1388622, which were previously found to be insignificant, have been identified by our new pipeline to be significantly associated with FA. Interestingly, *P2Y12* is the main receptor of ADP in platelets and a target of antiplatelet drugs prescribed to CVD patients [67]. *P2Y12* has been widely studied in order to understand its associated risks and devise better treatment strategies for CVDs [67–69], suggesting that these SNPs in this gene also have potential biological and clinical significance.

Moreover, our pipeline identified significant non-synonymous key SNP rs2071676 in *CA9*, which is associated with FA and was previously unidentified. The *CA9* product (CA IX) is one of the isoforms of the carbonic anhydrases which have been linked with several disease problems [70] in addition to the platelet and CVD [71]. Moreover, several *CA9* polymorphisms have been identified to be associated with oncological problems [72,73]. Thus, it might be worth pursuing the effects of the rs2071676 SNP that may underlie *CA9* with its product and platelet functions.

Furthermore, for the FC and PC platelet responses, the pipeline identified several common variants that are known to play key distinctive roles in the CRP-XL activated platelet responses. These variants include many of the previously identified *GP6* SNPs, such as rs41306982, non-synonymous rs1654439, rs1613662 and others in addition to rs3557 in *FCERIG*. Additionally, this study has identified SNPs rs748281, and rs41316468 in *PTK2B* to be significantly associated with FC and PC respectively which were previously unidentified. The *PTK2B* gene has also been implicated with blood pressure and hypertension [74], which further may suggest that these SNPs may be potential biomarkers for future studies involving platelets and CVDs, further underscoring the ability of our approach in identifying key SNPs.

### Conclusion

We have developed a robust computational tool for rapid discovery of key bio-markers associated with complex phenotypes. Here we have applied the approach to reveal previously unidentified SNPs associated with platelet response phenotypes that have been independently implicated in CVDs. This strongly suggests that our approach is robust in identifying key genetic variants or SNPs that are likely to be missed by following only the standard single method. Thus, our approach has strong potential to become a useful additional tool for rapid discovery of key important biomarkers prior to performing complex analyses in GASs. Moreover, it may be generally applied in other disease contexts for the discovery of multiple genetic variations that may better account for the heritability of continuous phenotypes.

R scripts codes used to run these analyses are in the S1 Text. The data supporting the findings are in S1 Dataset.

## Supporting information

S1 Table Consensus identification of the most significant SNPs associated with FC platelet response. (DOCX)

S2 Table Consensus identification of the most significant SNPs associated with PC platelet response. (DOCX)

S3 Table The frequency of each selected significant SNP associated with PA in each iteration. (XLSX)

S4 Table The frequency of each selected significant SNP associated with FA in each iteration. (XLSX)

S5 Table The frequency of each selected significant SNP associated with simulated phenotype in each iteration. (XLSX)

S6 Table The significance of the intermediate models due to the exclusion and inclusion of age as covariate for the PA platelet response. (DOCX)

S7 Table The frequency of each selected significant SNP associated with PA platelet response in each iteration. (DOCX)

S8 Table The frequency of each selected significant SNP associated with the FA response in each iteration. (DOCX)

S1 Text R script used in the analyses. (TXT)

S2 Text R script for reproducing the simulated data/results. (TXT)

S1 Dataset Supporting dataset. (CSV)

S1 File Additional supporting data. (XLS)

S1 Fig The residual plot when fitting the PA response using SNPs with or without age as a covariate. (TIF)

S2 Fig The residual plot when fitting the FA response using SNPs with or without age as a covariate. (TIF)

S3 Fig The frequency plot showing the overall selected significant SNPs that are associated with the PA platelet response in the intermediate models in all four iterations when age is included as a covariate. Most of the selected SNPs are similar to those selected when age is not included as a covariate.
(TIF)

S4 Fig The frequency plot showing the overall selected significant SNPs that are associated with the FA platelet response in the intermediate models in all four iterations when age is included as a covariate. (TIF)

S5 Fig The importance of the variables (SNPs and age), which have been selected by the RF based on their ranks and that are associated with FA. (TIF)
