# Supplementary material for: RAPIDSNPs: A new computational pipeline for rapidly identifying key genetic variants reveals previously unidentified SNPs that are significantly associated with individual platelet responses
Source: PLoS One. 2017 Apr 25;12(4):e0175957. doi: 10.1371/journal.pone.0175957 (PMC5404774; doi:10.1371/journal.pone.0175957)
Supplement: S1 Table — (DOCX) [file pone.0175957.s001.docx]

**S1 Table. Consensus identification of the most significant SNPs associated with FC platelet response**.

|  | | Stepwise (Jones et al 2009) | RF with Stepwise | RF with Ridge regression | RF with LASSO | RF with Boruta (P=0.01) | Consensus (3/5) |
| --- | --- | --- | --- | --- | --- | --- | --- |
| Platelet response type | | FC | FC | FC | FC | FC | FC |
| SNPs ID | Gene |  |  |  |  |  |  |
| rs41306982 | *GP6* | ✔ (<1x10-16) | ✔  (1.76e-08) | ✔  (5.25e-05) | ✔ (6.22e-09) | ✔ | ✔ |
| rs1613662 | *GP6* | ✔ (<1x10-16) | × | ✔ (0.0001) | × | ✔ | ✔ |
| rs3557 | *FCER1G* | ✔ (0.00001) | ✔ (0.006) | ✔ (0.0037) | ✔ (0.0048) | ✔ | ✔ |
| rs3737224 | *PEAR1* | ✔ (0.00026) | × | × | × | × |  |
| rs5746223 | *RAF1* | ✔ (0.0045) | × | × | × | × |  |
| rs1802141 | *LAT* | ✔ (0.006) | × | × | × | × |  |
| rs41307923 | *MAP2K4* | ✔ (0.003) | × | × | × | × |  |
| rs186295 | *VAV1* | ✔(0.007) | × | × | × | × |  |
| rs7806711 | *CD36* | (0.009) | × | × | × | × |  |
| rs7544500 | *CDC42* | ✔(0.009) | × | ✔ (0.0071) | × | ✔ | ✔ |
| rs41315906 | *ITGA2* | ×(0.108) | × | × | × | ✔ |  |
| rs6056043 | *PLCB1* | ×(0.190) | ✔  (0.0179) | × | × | ✔ |  |
| rs9823748 | *ITPR1* | ×(0.674) | ✔ (0.005) | × | ✔ (0.0003) | ✔ | ✔ |
| rs1057738 | *PRKACB* | × | ✔ (0.00703) | × | × | ✔ |  |
| rs748281 | *PTK2B* | × (0.844) | ✔ (0.0023) | ✔ (0.0025) | ✔ (0.0008) | × | ✔ |
| rs2555180 | *ILK* | ×(0.358) | × | ✔ (0.0068) | ✔ (0.01) | × |  |
| rs1654431 | *GP6* | ✔ (0.00002) | × | ✔ (0.0118) | × | × |  |
| rs28763993 | *PIK3CG* | ×(0.145) | × | × | ✔ (0.005) | × |  |
| rs2892811 | *PRKACB* | × | × | × | × | ✔ |  |
| rs11084382 | *GP6* | ✔ (<1x10-16) | × | × | × | ✔ |  |
| rs11264579 | *PEAR1* | ×(0.319) | × | × | × | ✔ |  |
| rs1654439 | *GP6* | ✔(<1x10-16) | × | × | × | ✔ |  |
| rs3809624 | *TBX6* | × | × | × | × | ✔ |  |
| rs822441 | *PEAR1* | ×(0.228) | × | × | × | ✔ |  |
| rs1671176 | *GP6* | ✔(<1x10-16) | × | × | × | ✔ |  |
| rs9604573 | *GAS6* | × | × | × | × | ✔ |  |
| rs1671218 | *GP6* | ✔(<1x10-16) | × | × | × | ✔ |  |
| rs1671214 | *GP6* | ✔(<1x10-16) | × | × | × | ✔ |  |
