# Supplementary material for: RAPIDSNPs: A new computational pipeline for rapidly identifying key genetic variants reveals previously unidentified SNPs that are significantly associated with individual platelet responses
Source: PLoS One. 2017 Apr 25;12(4):e0175957. doi: 10.1371/journal.pone.0175957 (PMC5404774; doi:10.1371/journal.pone.0175957)
Supplement: S2 Table — (DOCX) [file pone.0175957.s002.docx]

**S2 Table**

**Consensus identification of the most significant SNPs associated with PC platelet response.**

|  | | Stepwise (Jones et al 2009) | RF with Stepwise | RF with Ridge regression | RF with LASSO | RF with Boruta (P=0.01) | Consensus (3/5) |
| --- | --- | --- | --- | --- | --- | --- | --- |
| Platelet response type | | PC | PC | PC | PC | PC | PC |
| SNPs ID | Gene |  |  |  |  |  |  |
| rs41306982 | *GP6* | ✔(<1x10-16) | ✔  (2.83-15) | ✔  (1.07-07) | ✔ (< 2-16) | ✔ | ✔ |
| rs1613662 | *GP6* | ✔(<1x10-16) | × | ✔(2.70-07) | × | ✔ | ✔ |
| rs3557 | *FCER1G* | ✔ (0.0001) | × | × | × | × |  |
| rs41275750 | *AKT2* | ✔(0.002) | × | × | × | × |  |
| rs41307923 | *MAP2K4* | ✔(0.006) | × | × | × | × |  |
| rs1049654 | *CD36* | ✔(0.001) | × | × | × | × |  |
| rs41299597 | *PEAR1* | ✔(0.001) | × | × | × | × |  |
| rs6510959 | *NSR* | ✔(0.009) | × | × | × | × |  |
| rs4504857 | *CDC42* | ×(0.109) | ✔(0.00176) | ✔(0.0054) | ✔(0.005) | ✔ | ✔ |
| rs10424969 | *GP6* | ×(0.021) | ✔(0.0132) | ✔(0.0049) | × | ✔ | ✔ |
| rs7547892 | *PRKACB* | × | ✔ (0.0049) | ✔(0.0043) | ✔(0.0070) | × | ✔ |
| rs9384805 | *FYN* | ×(0.046) | ✔(0.0081) | ✔(0.0141) | ✔(0.0064) | × | ✔ |
| rs1654439 | *GP6* | ✔(<1x10-16) | × | ✔(0.018) | × | ✔ | ✔ |
| rs11084382 | *GP6* | ✔(<1x10-16) | × | ✔(0.0005) | × | ✔ | ✔ |
| rs41291724 | *GNAI2* | ×(0.058) | × | ✔(0.0163) | ✔(0.01) | × |  |
| rs28514576 | *TEC* | × (0.27) | × | × | ✔(0.0053) | × |  |
| rs17548850 | *PIK3CB* | × (0.273) | × | × | ✔(0.0184) | × |  |
| rs1654416 | *GP6* | ✔(<1x10-16) | × | × | × | ✔ |  |
| rs8191973 | *GAS6* | × | × | × | × | ✔ |  |
| rs1671150 | *GP6* | ✔(<1x10-16) | × | × | × | ✔ |  |
| rs2304167 | *GP6* | ✔(<1x10-16) | × | × | × | ✔ |  |
| rs17836542 | *GP6* | ✔(0.0006) | × | × | × | ✔ |  |
| rs1671153 | *GP6* | ✔(<1x10-16) | × | × | × | ✔ |  |
| rs2850161 | *PFKL* | × | × | × | × | ✔ |  |
| rs2124089 | *GP6* | ✔(0.00002) | × | × | × | ✔ |  |
| rs1654410 | *GP6* | ✔(0.0001) | × | × | × | ✔ |  |
| rs17836526 | *GP6* | ✔(0.001) | × | × | × | ✔ |  |
| rs1671218 | *GP6* | ✔(<1x10-16) | × | × | × | ✔ |  |
| rs4806637 | *GP6* | ✔(0.00004) | × | × | × | ✔ |  |
| rs41316468 | *PTK2B* | ×(0.445) | × | × | × | ✔ |  |
| rs1671214 | *GP6* | ✔(<1x10-16) | × | × | × | ✔ |  |
| rs2241655 | *PTK2B* | ×(0.949) | × | × | × | ✔ |  |
| rs1671176 | *GP6* | ✔(<1x10-16) | × | × | × | ✔ |  |
| rs41302194 | *MYLK* | ×(0.596) | × | × | × | ✔ |  |
| rs41271437 | *MYLK* | ×(0.475) | × | × | × | ✔ |  |
