# Supplementary material for: RAPIDSNPs: A new computational pipeline for rapidly identifying key genetic variants reveals previously unidentified SNPs that are significantly associated with individual platelet responses
Source: PLoS One. 2017 Apr 25;12(4):e0175957. doi: 10.1371/journal.pone.0175957 (PMC5404774; doi:10.1371/journal.pone.0175957)
Supplement: S6 Table — (DOCX) [file pone.0175957.s006.docx]

**S6 Table**

**The significance of the intermediate models due to the exclusion and inclusion of age as covariate to the PA platelet response.**

| #Iterations | Intermediate models with no age covariate | | | Intermediate models with age covariate | | |
| --- | --- | --- | --- | --- | --- | --- |
|  | RF+Stepwise | RF+Ridge regression | RF+Lasso | RF+Stepwise | RF+Ridge regression | RF+Lasso |
|  | Model significance (r-squared & p-values) | Model significance (%Variance) | Model significance (r-squared & p-values) | Model significance (r-squared & p-values) | Model significance (%Variance) | Model significance (r-squared & p-values) |
| 1 | 0.088 & 4.965e-09 | 14.7 | 0.096 & 1.83e-09 | 0.09  & 4.06e-09 | 18.21 | 0.097  & 1.38e-09 |
| 2 | 0.11 & 4.771e-11 | 17.83 | 0.10 & 8.1e-10 | 0.089  & 4.136e-09 | 18.01 | 0.097  & 2.63e-09 |
| 3 | 0.111 & 5.4e-11 | 18.77 | 0.138 & 7.108e-13 | 0.123  & 6.982e-12 | 15.92 | 0.12  & 2.98e-11 |
| 4 | 0.13 & 1.49e-12 | 17.5 | 0.16 & 6.61e-15 | 0.113  & 3.286e-11 | 13.53 | 0.096  & 8.64e-10 |
