# Supplementary material for: RAPIDSNPs: A new computational pipeline for rapidly identifying key genetic variants reveals previously unidentified SNPs that are significantly associated with individual platelet responses
Source: PLoS One. 2017 Apr 25;12(4):e0175957. doi: 10.1371/journal.pone.0175957 (PMC5404774; doi:10.1371/journal.pone.0175957)
Supplement: S7 Table — (DOCX) [file pone.0175957.s007.docx]

**S7 Table**

**The frequency of each selected significant SNP associated with PA platelet response in each iteration**

| SNP’s frequency of appearance in the models | SNP’s Id | Iteration number | RF + Model Name |
| --- | --- | --- | --- |
| 1 | rs3212391 | 1 | Stepwise |
| 2 | rs6141803 | 1 | Stepwise |
| 3 | rs2300065 | 1 | Stepwise |
| 4 | rs6442895 | 1 | Stepwise |
| 5 | rs12592919 | 1 | Stepwise |
| 6 | rs12709458 | 1 | Ridge |
| 7 | rs12592919 | 1 | Ridge |
| 8 | rs6442896 | 1 | Ridge |
| 9 | rs6136 | 1 | Lasso |
| 10 | rs2300065 | 1 | Lasso |
| 11 | rs927239 | 1 | Lasso |
| 12 | rs12592919 | 1 | Lasso |
| 13 | rs6442896 | 1 | Lasso |
| 14 | rs3212391 | 1 | Lasso |
| 15 | rs3212391 | 1 | Boruta |
| 16 | rs6141803 | 1 | Boruta |
| 17 | rs3212386 | 1 | Boruta |
| 18 | rs16865105 | 1 | Boruta |
| 19 | rs6442895 | 1 | Boruta |
| 20 | rs2424895 | 1 | Boruta |
| 21 | rs2424905 | 1 | Boruta |
| 22 | rs6442896 | 1 | Boruta |
| 23 | rs6433658 | 1 | Boruta |
| 24 | rs17041401 | 1 | Boruta |
| 25 | rs13316843 | 1 | Boruta |
| 26 | rs12592919 | 1 | Boruta |
| 27 | rs26682 | 2 | Stepwise |
| 28 | rs2292867 | 2 | Stepwise |
| 29 | rs6141803 | 2 | Stepwise |
| 30 | rs6442895 | 2 | Stepwise |
| 31 | rs1527480 | 2 | Stepwise |
| 32 | rs6442896 | 2 | Ridge |
| 33 | rs6442895 | 2 | Ridge |
| 34 | rs2292867 | 2 | Ridge |
| 35 | rs3745406 | 2 | Ridge |
| 36 | rs1527480 | 2 | Ridge |
| 37 | rs6141803 | 2 | Lasso |
| 38 | rs12592919 | 2 | Lasso |
| 39 | rs7187863 | 2 | Lasso |
| 40 | rs1527480 | 2 | Lasso |
| 41 | rs10061730 | 2 | Lasso |
| 42 | rs3745406 | 2 | Lasso |
| 43 | rs2292867 | 2 | Lasso |
| 44 | rs6141803 | 2 | Boruta |
| 45 | rs6442895 | 2 | Boruta |
| 46 | rs6442896 | 2 | Boruta |
| 47 | rs6433658 | 2 | Boruta |
| 48 | rs17041401 | 2 | Boruta |
| 49 | rs12592919 | 2 | Boruta |
| 50 | rs26682 | 2 | Boruta |
| 51 | rs10061730 | 2 | Boruta |
| 52 | rs3212386 | 2 | Boruta |
| 53 | rs2424905 | 2 | Boruta |
| 54 | rs2424895 | 2 | Boruta |
| 55 | rs1527480 | 3 | Stepwise |
| 56 | rs3212391 | 3 | Stepwise |
| 57 | rs3730051 | 3 | Stepwise |
| 58 | rs6442896 | 3 | Stepwise |
| 59 | rs6141803 | 3 | Stepwise |
| 60 | rs2300065 | 3 | Stepwise |
| 61 | rs11637556 | 3 | Stepwise |
| 62 | rs6141803 | 3 | Ridge |
| 63 | rs3730051 | 3 | Ridge |
| 64 | rs6442896 | 3 | Ridge |
| 65 | rs1527480 | 3 | Ridge |
| 66 | rs11637556 | 3 | Ridge |
| 67 | rs6442895 | 3 | Ridge |
| 68 | rs6141803 | 3 | Lasso |
| 69 | rs3730051 | 3 | Lasso |
| 70 | rs6442896 | 3 | Lasso |
| 71 | rs1527480 | 3 | Lasso |
| 72 | rs2300065 | 3 | Lasso |
| 73 | rs3212386 | 3 | Lasso |
| 74 | rs11637556 | 3 | Lasso |
| 75 | rs6141803 | 3 | Boruta |
| 76 | rs6442895 | 3 | Boruta |
| 77 | rs6442896 | 3 | Boruta |
| 78 | rs6433658 | 3 | Boruta |
| 79 | rs17041401 | 3 | Boruta |
| 80 | rs3212391 | 3 | Boruta |
| 81 | rs17760545 | 3 | Boruta |
| 82 | rs2424895 | 3 | Boruta |
| 83 | rs7568033 | 3 | Boruta |
| 84 | rs3212418 | 3 | Boruta |
| 85 | rs26682 | 3 | Boruta |
| 86 | rs33443 | 3 | Boruta |
| 87 | rs246410 | 3 | Boruta |
| 88 | rs397454 | 3 | Boruta |
| 89 | rs2424905 | 3 | Boruta |
| 90 | rs16865105 | 3 | Boruta |
| 91 | rs6442896 | 4 | Stepwise |
| 92 | rs3212391 | 4 | Stepwise |
| 94 | rs3730051 | 4 | Stepwise |
| 95 | rs6141803 | 4 | Stepwise |
| 96 | rs1527480 | 4 | Stepwise |
| 97 | rs11637556 | 4 | Stepwise |
| 98 | rs6442896 | 4 | Ridge |
| 99 | rs3730051 | 4 | Ridge |
| 100 | rs1527480 | 4 | Ridge |
| 101 | rs11637556 | 4 | Ridge |
| 102 | rs6442896 | 4 | Lasso |
| 103 | rs3730051 | 4 | Lasso |
| 104 | rs11637556 | 4 | Lasso |
| 105 | rs3212418 | 4 | Lasso |
| 106 | rs6141803 | 4 | Lasso |
| 107 | rs6442896 | 4 | Boruta |
| 108 | rs6433658 | 4 | Boruta |
| 109 | rs6442895 | 4 | Boruta |
| 110 | rs3212391 | 4 | Boruta |
| 111 | rs17041401 | 4 | Boruta |
| 112 | rs6058869 | 4 | Boruta |
| 113 | rs33443 | 4 | Boruta |
| 114 | rs6895049 | 4 | Boruta |
| 115 | rs2424895 | 4 | Boruta |
| 116 | rs26682 | 4 | Boruta |
| 117 | rs3212418 | 4 | Boruta |
| 118 | rs2424905 | 4 | Boruta |
| 119 | rs7568033 | 4 | Boruta |
| 120 | rs16865105 | 4 | Boruta |
