# Supplementary material for: RAPIDSNPs: A new computational pipeline for rapidly identifying key genetic variants reveals previously unidentified SNPs that are significantly associated with individual platelet responses
Source: PLoS One. 2017 Apr 25;12(4):e0175957. doi: 10.1371/journal.pone.0175957 (PMC5404774; doi:10.1371/journal.pone.0175957)
Supplement: S8 Table — (DOCX) [file pone.0175957.s008.docx]

**S8 Table**

**The frequency of each selected significant SNP associated with the FA response in each iteration.**

| SNP’s frequency of appearance in the models | SNP’s Id | Iteration number | RF + Model Name |
| --- | --- | --- | --- |
| 1 | rs1388622 | 1 | Stepwise |
| 2 | rs11637556 | 1 | Stepwise |
| 3 | rs8192827 | 1 | Stepwise |
| 4 | rs1537593 | 1 | Stepwise |
| 5 | rs11637556 | 1 | Ridge |
| 6 | rs7180408 | 1 | Ridge |
| 7 | rs1038639 | 1 | Ridge |
| 8 | rs11637556 | 1 | Lasso |
| 9 | rs1537593 | 1 | Lasso |
| 10 | rs1388622 | 1 | Lasso |
| 11 | rs3173798 | 1 | Boruta |
| 12 | rs1491978 | 1 | Boruta |
| 13 | rs11637556 | 1 | Boruta |
| 14 | rs1388622 | 1 | Boruta |
| 15 | rs17204437 | 1 | Boruta |
| 16 | rs1537593 | 1 | Boruta |
| 17 | rs9641866 | 1 | Boruta |
| 18 | rs1388622 | 2 | Stepwise |
| 19 | rs11637556 | 2 | Stepwise |
| 20 | rs7180408 | 2 | Stepwise |
| 21 | rs2290890 | 2 | Stepwise |
| 22 | rs11637556 | 2 | Ridge |
| 23 | rs7806711 | 2 | Ridge |
| 24 | rs9895150 | 2 | Ridge |
| 25 | rs7180408 | 2 | Ridge |
| 26 | rs11637556 | 2 | Lasso |
| 27 | rs1388622 | 2 | Lasso |
| 28 | rs2290890 | 2 | Lasso |
| 29 | rs11772036 | 2 | Lasso |
| 30 | rs41305272 | 2 | Lasso |
| 31 | rs9895150 | 2 | Lasso |
| 32 | rs1388622 | 2 | Boruta |
| 33 | rs11637556 | 2 | Boruta |
| 34 | rs6787801 | 2 | Boruta |
| 35 | rs1491978 | 2 | Boruta |
| 36 | rs1491978 | 3 | Stepwise |
| 37 | rs11637556 | 3 | Stepwise |
| 38 | rs10974955 | 3 | Stepwise |
| 39 | rs10499858 | 3 | Stepwise |
| 40 | rs2071676 | 3 | Stepwise |
| 41 | rs41282607 | 3 | Stepwise |
| 42 | rs1866047 | 3 | Stepwise |
| 43 | rs2071676 | 3 | Ridge |
| 44 | rs11637556 | 3 | Ridge |
| 45 | rs41282607 | 3 | Ridge |
| 46 | rs3212417 | 3 | Ridge |
| 47 | rs11637556 | 3 | Lasso |
| 48 | rs2071676 | 3 | Lasso |
| 49 | rs10974955 | 3 | Lasso |
| 50 | rs10499858 | 3 | Lasso |
| 51 | rs41282607 | 3 | Lasso |
| 52 | rs1866047 | 3 | Lasso |
| 53 | rs8192827 | 3 | Lasso |
| 54 | rs3736101 | 3 | Lasso |
| 55 | rs1491978 | 3 | Boruta |
| 56 | rs7034539 | 3 | Boruta |
| 57 | rs10974955 | 3 | Boruta |
| 58 | rs3173798 | 3 | Boruta |
| 59 | rs1537593 | 3 | Boruta |
| 60 | rs17204437 | 3 | Boruta |
| 61 | rs10499858 | 3 | Boruta |
| 62 | rs1388622 | 4 | Stepwise |
| 63 | rs11637556 | 4 | Stepwise |
| 64 | Age | 4 | Stepwise |
| 65 | rs2071676 | 4 | Stepwise |
| 66 | rs7034539 | 4 | Stepwise |
| 67 | rs2296275 | 4 | Stepwise |
| 68 | rs11772036 | 4 | Stepwise |
| 69 | rs11637556 | 4 | Ridge |
| 70 | rs7034539 | 4 | Ridge |
| 71 | rs1038639 | 4 | Ridge |
| 72 | rs2071676 | 4 | Ridge |
| 73 | rs11637556 | 4 | Lasso |
| 74 | rs3736101 | 4 | Lasso |
| 75 | rs2071676 | 4 | Lasso |
| 76 | rs11772036 | 4 | Lasso |
| 77 | rs1038639 | 4 | Lasso |
| 78 | rs304076 | 4 | Lasso |
| 79 | rs11637556 | 4 | Boruta |
| 80 | rs1491978 | 4 | Boruta |
| 81 | rs11772036 | 4 | Boruta |
| 82 | rs6787801 | 4 | Boruta |
| 83 | rs1388622 | 4 | Boruta |
| 84 | rs9641866 | 4 | Boruta |
