# Supplementary figures and images for: RAPIDSNPs: A new computational pipeline for rapidly identifying key genetic variants reveals previously unidentified SNPs that are significantly associated with individual platelet responses
Source: PLoS One. 2017 Apr 25;12(4):e0175957. doi: 10.1371/journal.pone.0175957 (PMC5404774; doi:10.1371/journal.pone.0175957)

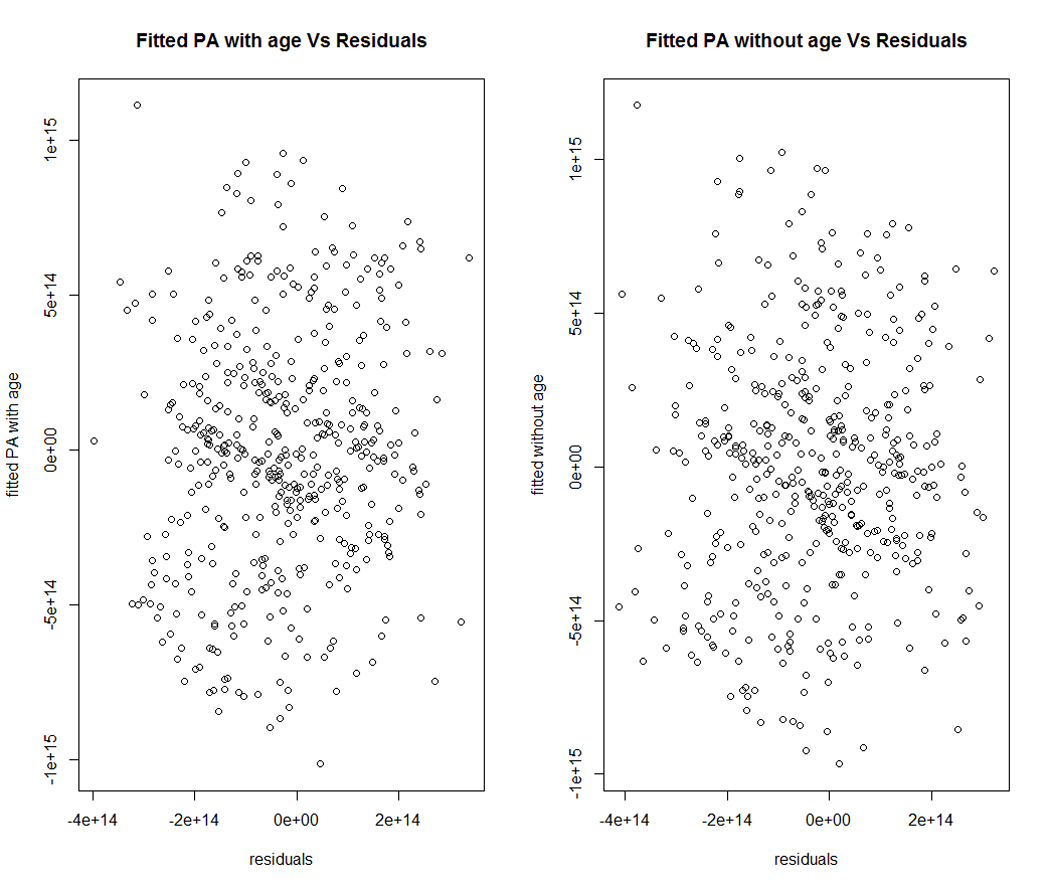

Supplement: S1 Fig — (TIF) [file pone.0175957.s013.tif]

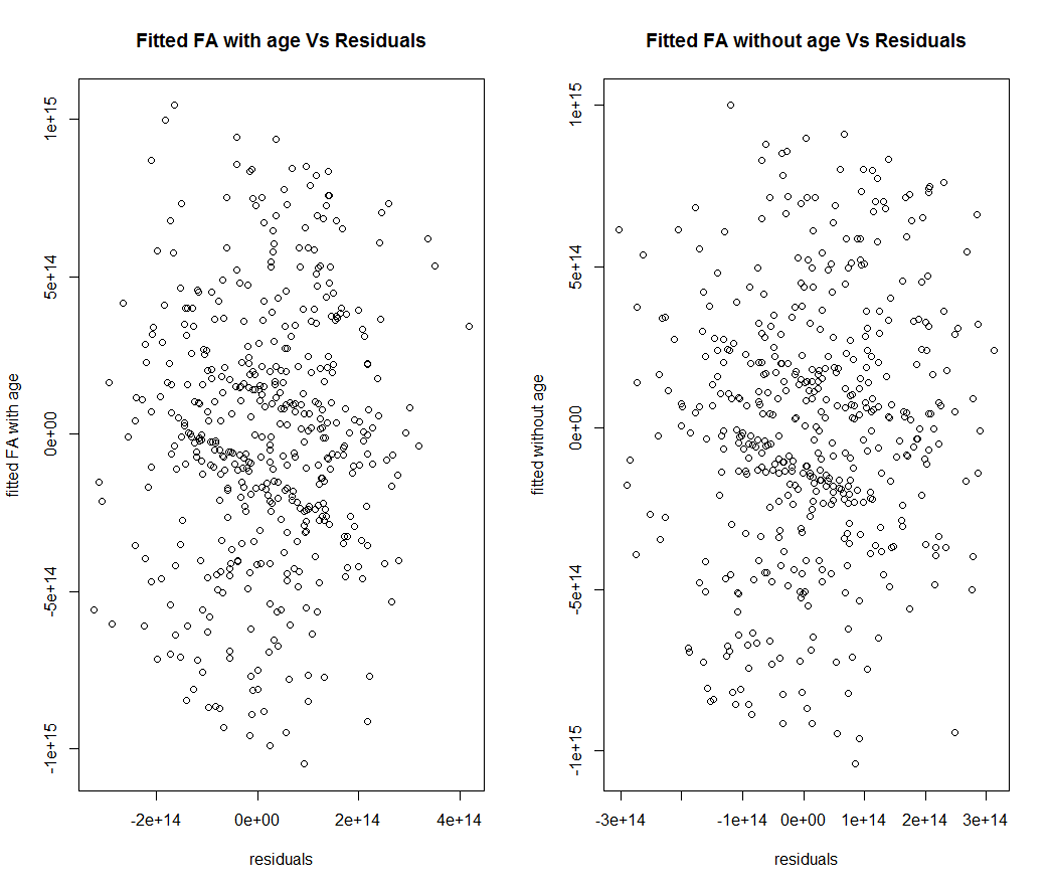

Supplement: S2 Fig — (TIF) [file pone.0175957.s014.tif]

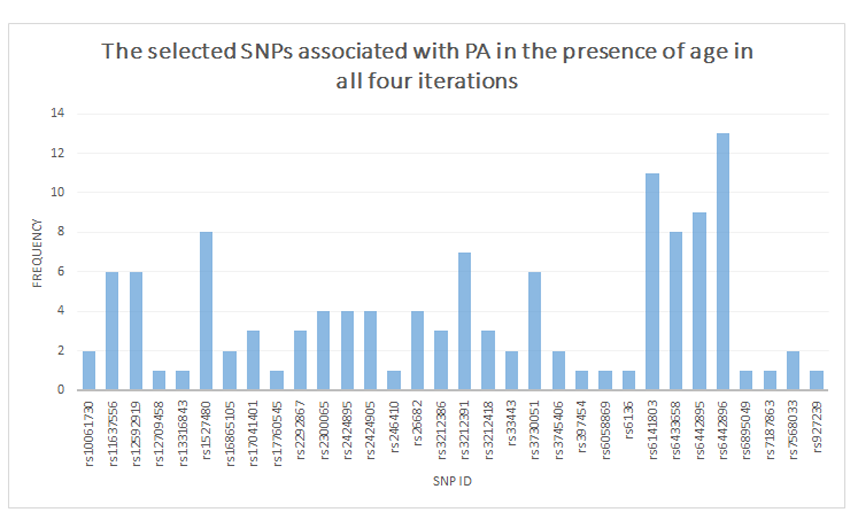

Supplement: S3 Fig — Most of the selected SNPs are similar to those selected when age is not included as a covariate. (TIF) [file pone.0175957.s015.tif]

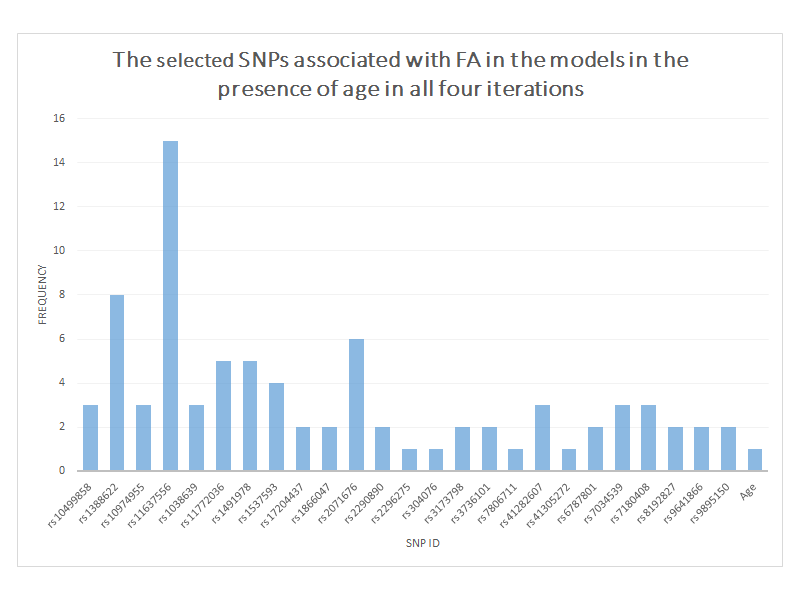

Supplement: S4 Fig — (TIF) [file pone.0175957.s016.tif]

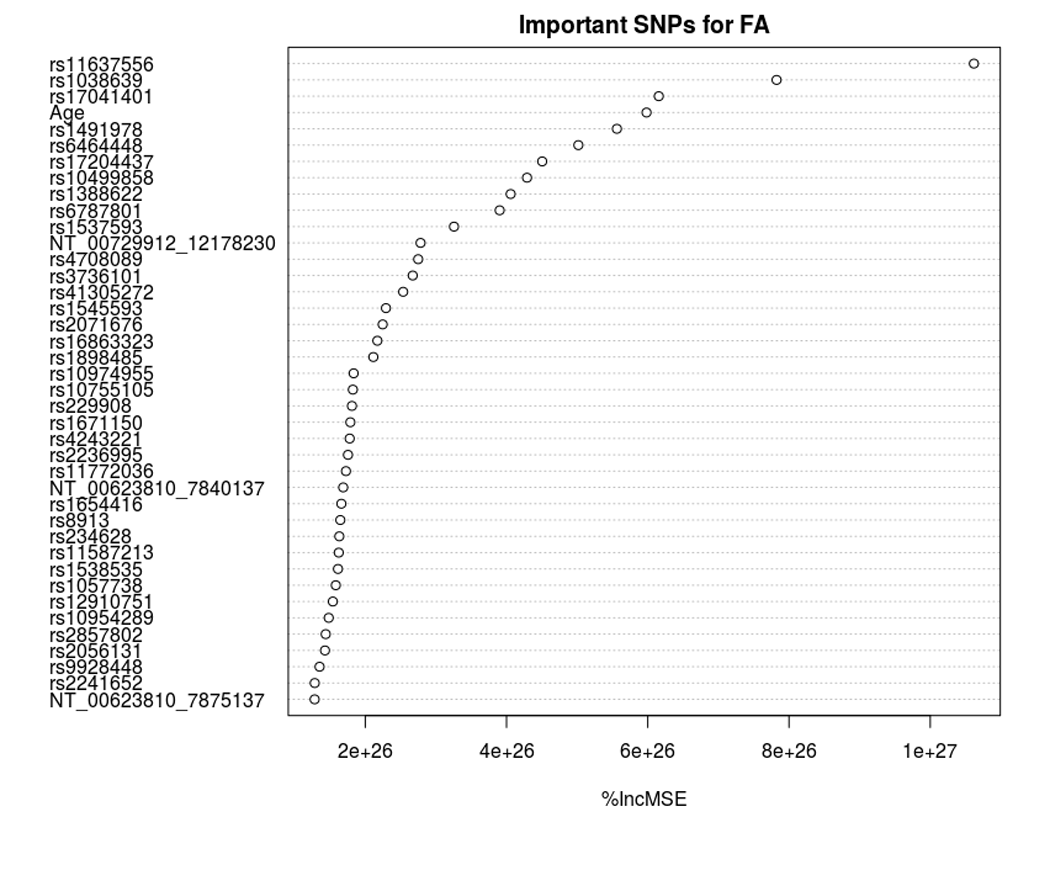

Supplement: S5 Fig — (TIF) [file pone.0175957.s017.tif]
